# Longitudinal changes in hippocampal texture from healthy aging to Alzheimer’s disease

**DOI:** 10.1093/braincomms/fcad195

**Published:** 2023-07-05

**Authors:** Alfie Wearn, Lars Lau Raket, D Louis Collins, R Nathan Spreng, for the Alzheimer’s Disease Neuroimaging Initiative

**Affiliations:** Department of Neurology and Neurosurgery, Montreal Neurological Institute, McGill University, Montreal, QC, Canada H3A 2B4; Clinical Memory Research Unit, Department of Clinical Sciences, Lund University, Lund SE-221 00, Sweden; Novo Nordisk A/S, Søborg 2860, Denmark; Department of Neurology and Neurosurgery, Montreal Neurological Institute, McGill University, Montreal, QC, Canada H3A 2B4; McConnell Brain Imaging Centre, McGill University, Montreal, QC, Canada H3A 2B4; Department of Neurology and Neurosurgery, Montreal Neurological Institute, McGill University, Montreal, QC, Canada H3A 2B4; McConnell Brain Imaging Centre, McGill University, Montreal, QC, Canada H3A 2B4; Departments of Psychology and Psychiatry, McGill University, Montreal, QC, Canada H3A 2B4; Douglas Mental Health University Institute, Verdun, QC, Canada H4H 1R3

**Keywords:** Alzheimer’s disease, MRI, texture analysis, radiomics, longitudinal modelling

## Abstract

Early detection of Alzheimer’s disease is essential to develop preventive treatment strategies. Detectible change in brain volume emerges relatively late in the pathogenic progression of disease, but microstructural changes caused by early neuropathology may cause subtle changes in the MR signal, quantifiable using texture analysis. Texture analysis quantifies spatial patterns in an image, such as smoothness, randomness and heterogeneity. We investigated whether the MRI texture of the hippocampus, an early site of Alzheimer’s disease pathology, is sensitive to changes in brain microstructure before the onset of cognitive impairment. We also explored the longitudinal trajectories of hippocampal texture across the Alzheimer’s continuum in relation to hippocampal volume and other biomarkers. Finally, we assessed the ability of texture to predict future cognitive decline, over and above hippocampal volume. Data were acquired from the Alzheimer’s Disease Neuroimaging Initiative. Texture was calculated for bilateral hippocampi on 3T T_1_-weighted MRI scans. Two hundred and ninety-three texture features were reduced to five principal components that described 88% of total variance within cognitively unimpaired participants. We assessed cross-sectional differences in these texture components and hippocampal volume between four diagnostic groups: cognitively unimpaired amyloid-β^−^ (*n* = 406); cognitively unimpaired amyloid-β^+^ (*n* = 213); mild cognitive impairment amyloid-β^+^ (*n* = 347); and Alzheimer’s disease dementia amyloid-β^+^ (*n* = 202). To assess longitudinal texture change across the Alzheimer’s continuum, we used a multivariate mixed-effects spline model to calculate a ‘disease time’ for all timepoints based on amyloid PET and cognitive scores. This was used as a scale on which to compare the trajectories of biomarkers, including volume and texture of the hippocampus. The trajectories were modelled in a subset of the data: cognitively unimpaired amyloid-β^−^ (*n* = 345); cognitively unimpaired amyloid-β^+^ (*n* = 173); mild cognitive impairment amyloid-β^+^ (*n* = 301); and Alzheimer’s disease dementia amyloid-β^+^ (*n* = 161). We identified a difference in texture component 4 at the earliest stage of Alzheimer’s disease, between cognitively unimpaired amyloid-β^−^ and cognitively unimpaired amyloid-β^+^ older adults (Cohen’s *d* = 0.23, *P*_adj_ = 0.014). Differences in additional texture components and hippocampal volume emerged later in the disease continuum alongside the onset of cognitive impairment (*d* = 0.30–1.22, *P*_adj_ < 0.002). Longitudinal modelling of the texture trajectories revealed that, while most elements of texture developed over the course of the disease, noise reduced sensitivity for tracking individual textural change over time. Critically, however, texture provided additional information than was provided by volume alone to more accurately predict future cognitive change (*d* = 0.32–0.63, *P*_adj_ < 0.0001). Our results support the use of texture as a measure of brain health, sensitive to Alzheimer’s disease pathology, at a time when therapeutic intervention may be most effective.

## Teaser

Wearn *et al*. show that texture analysis of the hippocampus can identify signs of Alzheimer’s disease pathology in cognitively unimpaired at-risk older adults, and can do so before differences in hippocampal volume are detectible. Hippocampal texture analysis can extract additional information from existing clinical MRI pipelines to improve patient prognosis.

## Graphical Abstract

**Graphical Abstract:**
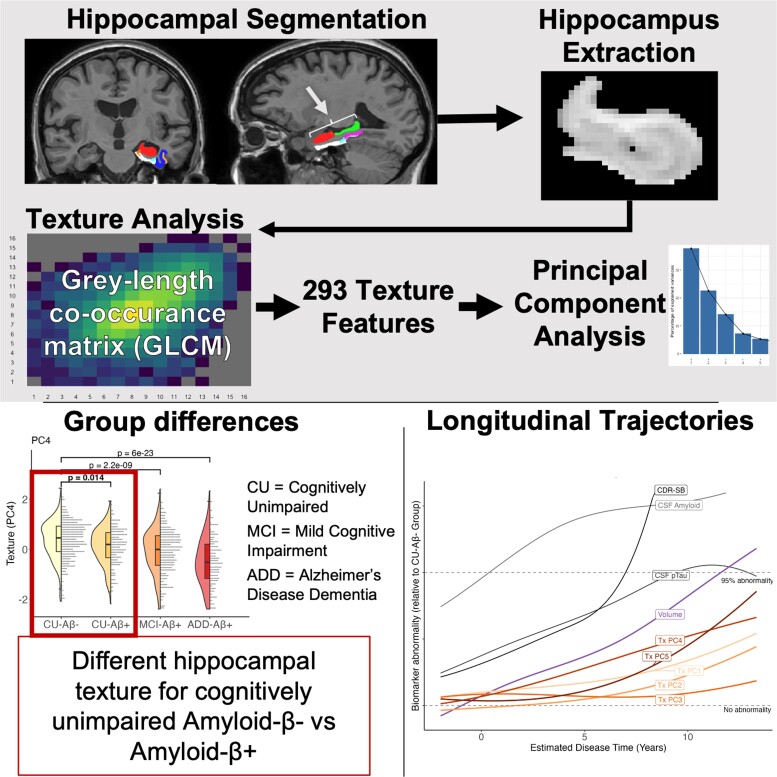

## Introduction

Early detection of Alzheimer’s disease is essential to develop effective preventive treatment strategies. Localized atrophy, measured as regional volume loss, is commonly detected using MRI and is used as an indicator of the pathology of Alzheimer’s disease.^1^ However, macroscopic volume loss is not a direct measure of the pathological hallmarks, rather, it is an indirect marker of the consequences of pathology. As a result, a detectible change in volume emerges relatively late in the pathogenic progression of disease^2^ and is likely to be irreversible.

Many features of the years-long preclinical phase of Alzheimer’s disease, including accumulation of neuritic plaques, formation of neurofibrillary tangles and neuroinflammation, begin to appear when pathology is less developed, and is therefore more likely to be therapeutically modifiable.^3^ The earliest known *in vivo* indicators of Alzheimer’s disease pathology is a decrease in the concentration of amyloid-beta (Aβ) in CSF, followed by localized increases in Aβ as detected using PET.^2^ These assays are invasive and the latter prohibitively expensive. MRI is a safer and relatively more accessible tool for clinical assessment that provides valuable spatial information. There is increasing evidence that microstructural changes caused by early neuropathology may cause subtle changes in the MR signal that can be quantified using texture analysis.^4–7^

Texture analysis, a branch of radiomics, exploits spatial patterns in an image, quantifying features such as smoothness, randomness and heterogeneity. Texture analysis of medical images has already provided promising results in the area of tumour classification^8^ (see review by Scalco and Rizzo^9^). More recently, texture analysis has been investigated as a tool for classifying, predicting and differentiating Alzheimer’s disease (reviewed by Cai *et al.*).^4^ Several studies have reported differences in brain MRI texture between people with Alzheimer’s disease and healthy older people^5,7,10,11^ and between people with mild cognitive impairment (MCI) and Alzheimer’s disease,^5,12^ and texture differences have been shown to predict future conversion of people with MCI to a diagnosis of Alzheimer’s disease.^5,7,10,13^ Texture differences can also be used to detect the presence of Aβ pathology (measured using PET) in people with MCI.^14^ Scoring using Non-local Image Patch Estimators (SNIPEs) is a method defined by patch intensity, contrast and texture, which has also been shown to be sensitive to Alzheimer’s disease pathology^15^ and predict conversion over time.^16,17^

A direct analysis of texture from structural MRI has the potential to detect subtle brain changes associated with Alzheimer’s disease pathology before a diagnosis of dementia. However, no research to date has examined whether it can detect pathologically relevant information in those who remain cognitively unimpaired (CU) but who have evidence of Aβ pathology, a known risk factor for Alzheimer’s disease. A primary aim of this study was to identify whether the texture of a standard structural MRI scan of the hippocampus, an early site of amyloid pathology in Alzheimer’s disease,^18^ is sensitive to such presymptomatic changes in the brain microstructure. Furthermore, all related studies to date have analysed only cross-sectional texture measures, so there currently exists no description of how texture develops within individuals over the disease course. Therefore, a second aim of this study was to explore how hippocampal texture changes across the Alzheimer’s disease continuum in a mixed cross-sectional/longitudinal design, particularly in relation to hippocampal volume and other biomarkers. Finally, we assessed to what extent measuring texture features could increase the value of clinical MRI scans by providing additional independent information on brain health to that provided by volumetry alone.

## Materials and methods

### Participants

Data used in the preparation of this article were obtained from the Alzheimer’s Disease Neuroimaging Initiative (ADNI) database (adni.loni.usc.edu). The ADNI was launched in 2003 as a public–private partnership, led by Principal Investigator Michael W. Weiner, MD. The primary goal of ADNI has been to test whether serial MRI, PET, other biological markers, and clinical and neuropsychological assessment can be combined to measure the progression of MCI and early Alzheimer’s disease. For all ADNI participants, written informed consent was acquired before procedures were performed in accordance with the Declaration of Helsinki. All ADNI studies were approved by the appropriate review boards prior to data collection.

**Figure 1:**
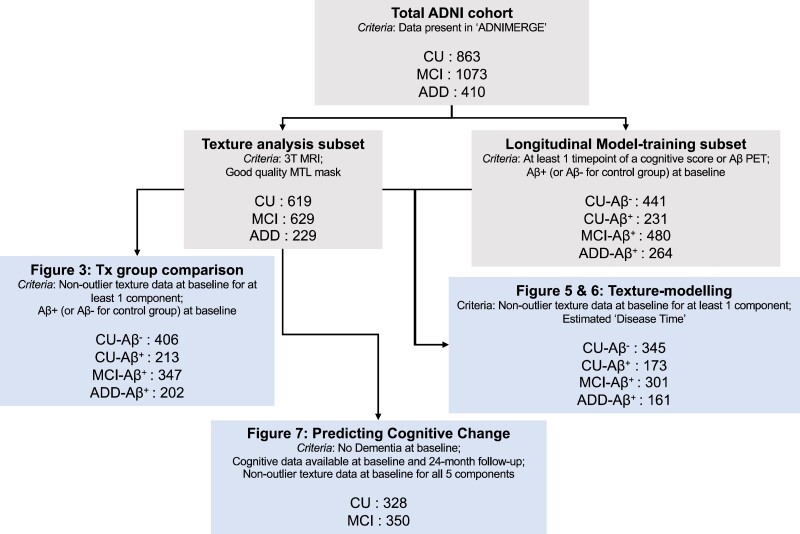
**Sample size of cohort subsets used in different analyses.** Inclusion criteria for participants in each subset are shown. Where amyloid status is relevant to the analysis, the cohort is described by four diagnostic groups instead of three, where the CU group is split into Aβ− (CU-Aβ^−^) and Aβ+ (CU-Aβ^+^) subgroups. In these cases, MCI and ADD groups are also Aβ+. ‘Cognitive tests’ refer to the 13-item ADAS-cog, CDR-SB and MMSE. Aβ PET data include ^18^F-Florbetapen Amyloid PET tracer and AV45 tracer data. ADAS13, Alzheimer’s Disease Assessment Scale (13 questions); CDR-SB, Clinical Dementia Rating scale Sum of Boxes; MMSE, Mini Mental State Examination.

The total sample included 863 (CU; age: 72.5 ± 6.56; years of education: 16.5 ± 2.53; 56.2% female), 1073 MCI (age: 72.8 ± 7.59; years of education: 16.0 ± 2.77; 41.3% female) and 410 Alzheimer’s disease dementia (ADD) participants (age: 74.8 ± 7.90; years of education: 15.2 ± 2.90; 43.4% female). Subsets of this total were used in different analyses, which will be reported in the relevant sections, and are summarized in Fig. 1. Two primary analysis subsets are reported below. A full list of subject IDs included in each cohort is available in the associated GitHub repository.

#### Texture analysis subset

Given the sensitivity of texture features to signal-to-noise, an *a priori* decision was made to calculate texture features only in data collected on 3 T MRI. Therefore, only participants from ADNIGO, two and three were selected for texture analysis. Furthermore, a total of 382 sessions across 214 participants were excluded due to region of interest (ROI) masks of insufficient quality (see ‘Image processing’ section below). This number is reasonably high as we employed strict criteria on mask quality, particularly at the boundaries, more so than might be necessary if studying volume alone. Texture analysis was therefore conducted on a subset of the complete data that comprised 619 CU (age: 72.2 ± 6.28), 629 MCI (age: 72.0 ± 7.37) and 229 ADD participants (age: 73.7 ± 7.85).

#### Longitudinal model training subset

Estimating the continuous measure of ‘disease time’ required participants to have a determined amyloid status (see ‘CSF biomarkers’ section below) and data for any of 3 cognitive tests [13-item ADAS-cog, Clinical Dementia Rating Scale Sum of Boxes (CDR-SB) and Mini Mental State Examination (MMSE)] or amyloid PET, either using the AV45 tracer or ^18^F-Florbetapen Amyloid PET tracer. One participant was excluded for having extremely high AV45 values compared with the rest of sample. These estimated disease times were therefore calculated for a subset of the complete data (including ADNI1) that comprised 1416 participants: 441 CU-Aβ^−^ (age: 71.6 ± 6.20), 231 CU-Aβ^+^ (age: 73.4 ± 6.33), 480 MCI-Aβ^+^ (age: 73.2 ± 7.09) and 264 ADD-Aβ^+^ (age: 74.2 ± 7.94).

### CSF biomarkers

Aβ was used as a grouping factor for cross-sectional analysis. Participants were defined as ‘Aβ^+^’ based on Aβ_1–42_ thresholds provided by ADNI. Where CSF data were available, a threshold of 980 pg/mL was used,^19^ as measured using the Roche Elecsys immunoassay platform. Where CSF data were not available for a given participant, amyloid PET data were used at a Centiloid threshold.^20^ Centiloids are a method used to consolidate PET data from multiple radiotracers, in this case, AV45 and ^18^F-Florbetapen Amyloid PET tracer, and to mitigate inter-site variability.^21^ Participants were excluded if the values fell within 5% of these defined boundaries in order to mitigate noise due to easily misclassified borderline cases, as has been employed in previous studies^22–24^ (a total of 103 subjects in the total ADNI cohort). CSF concentrations of phosphorylated tau (pTau) were explored in multivariate longitudinal modelling of biomarker trajectories.

### Imaging data

T_1_-weighted structural MRI data for participants in phases ADNIGO, two and three (as these data were collected at 3 T) were downloaded from adni.loni.usc.edu. Clinical information, cognitive test scores and biomarker data were extracted from the ‘ADNIMERGE’ datasheet, downloaded 25 March 2022. Gradwarp, B1-inhomogeneity corrected and N3 bias field corrected images were converted to Brain Imaging Data Structure (BIDS) format using the Clinica ‘ADNI-to-BIDS’ pipeline.^25^

### Image processing

Left and right hippocampi were segmented in native space using the T_1_-only routine of Automatic Segmentation of Hippocampal Subfields, using UPENN-PMC atlas dated 20 July 2018.^26^ Masks were visually inspected for quality, and excluded if the hippocampal ROI overlapped with surrounding CSF or non-hippocampal structures, or missed parts of the hippocampus. This atlas separately segments anterior and posterior hippocampal subregions, which for this study were combined to create a whole hippocampus ROI which was used to mask the T1w scans (using *fslmaths*).^27^

The MRI intensities within the extracted ROIs were denoised using Advanced Normalization Tools DenoiseImage (which did not significantly affect the characterization of texture features).^28^ Extreme high and low values were then excluded from denoised ROIs using the μ ± 3σ technique described by Collewet *et al*.^29^ This was done to limit the effects of partial voluming from surrounding CSF or other tissues. Finally, ROIs were *z*-scored to normalize intensity across participants, as recommended by Um *et al*.^30^

#### Texture analysis

Texture analysis was conducted using the Radiomics Image Analysis R package.^31^ First-order statistics, 3D grey-level co-occurrence matrices and grey-level run-length matrices were computed for the left and right hippocampus. In brief, each element is described as follows. First-order statistics describe the shape of the histogram of signal across the region and includes measures such as skew, kurtosis, energy and entropy. The grey-level co-occurrence matrices assess the spatial relationship between pairs of voxels to measure how often certain values appear adjacently (at a distance of one voxel) in each direction (all 26 directions were assessed and averaged). The grey-level run-length matrices assess how many same-value voxels appear adjacent to each other, that is, it is sensitive to runs of the same intensity. For grey-level co-occurrence matrices and grey-level run-length matrices, images intensities were discretized into 32 equal-sized bins. A total of 293 features, as well as volume, were computed. A full description of each of these features is provided by Kolossváry *et al*.^31^ Texture features (and volume) were averaged between hemispheres for all analyses.

Different texture analysis procedures and pre-processing pipelines can make a considerable difference to texture features and there is little consensus on best practices. Where possible, we have followed guidelines in the literature and attempted to clearly state our analysis decisions to improve replicability of our findings.^32–34^

### Statistical analysis

#### Feature reduction

We used principal component analysis as a feature reduction technique to broadly define texture. Extreme outliers (mean ± 3 × interquartile range) were excluded from texture variables (0.7% of all data points). Twenty-five variables with very low variance (<1e−10) were also excluded. Variables were *z*-scored before calculating the principal component analysis. The principal component analysis rotation was calculated only on baseline data from CU participants with complete texture data and then applied to the entire dataset. We aimed to study all components that described ≥5% of the variance across CU participants.

#### Cross-sectional analyses

Linear mixed-effects models were used to assess the relationship between diagnostic group at baseline and hippocampal texture and volume in order to determine at which disease stage each measure is detectibly different to the healthy state:


(1) TextureorVolume∼Dx_bl+Age_bl+Sex+Education+ICV+(1|Site).

One model was run for each texture component or volume as the dependent variable. Four baseline diagnostic groups (*Dx_bl*, *a categorical variable*) were assessed, defined by a combination of clinical diagnosis and amyloid status: CU-Aβ^−^, CU-Aβ^+^, MCI-Aβ^+^ and ADD-Aβ^+^. Models were corrected for baseline age (*Age_bl*), sex, years of education, intracranial volume and scan site. Three pairwise *post hoc* models for each of the six variables were also made comparing the Healthy group to each other group. *P*-values shown for this analysis are adjusted for multiple comparisons across all 18 tests using Benjamini–Holm method of false discovery correction. Cohen’s *f* and *d* effect sizes were computed. Outliers, defined as mean ± 1.5 × interquartile range, were excluded from these analyses.

#### Disease progression modelling

Longitudinal change in brain structure can be assessed between groups across various scales including age, time since baseline, or even cognitive impairment; however, it is difficult to define age at disease *onset* for subjects in the ADNI database. This is necessary to be able to merge CU, MCI and Alzheimer’s disease subjects together on a common timeline to see how texture features change with disease progression. To assess longitudinal texture change across the Alzheimer’s disease continuum, we used a novel approach to estimate a ‘disease time’ along which participant timepoints could be staged. This method is based on an approach described and validated by Kühnel *et al*.,^35^ and Raket,^36^ available in the *progmod* R package (github.com/larslau/progmod). The method shares the basic assumption of a single disease trajectory along a latent disease time scale with related methods such as the GRowth models by Alternating Conditional Expectation and Latent-Time Joint Mixed-effects Models. Compared with these methods, our method differs by using data on its original scale and estimating different variance parameters for different outcomes by maximum likelihood estimation, enabling a data-driven relative weighting of different outcome measures. Furthermore, the methods differ in terms of the parametrization of the mean trajectories and patient-level deviations; of note is that our method does not allow patient-level random slopes to model deviations from the mean. These choices have previously been shown to give better performance for predicting future trajectories of patients on the Alzheimer’s disease continuum compared with GRowth models by Alternating Conditional Expectation and Latent-Time Joint Mixed-effects Models.^35^

##### Estimating a latent disease timeline

Here, we simultaneously model the multivariate trajectory of the amyloid centiloid score (PET) and three cognitive test scores: Alzheimer’s Disease Assessment Scale (13-item version; ADAS13), CDR-SB and MMSE to place each subject on a common time scale.

Disease progression was modelled with a nonlinear mixed-effects model that jointly described the trajectories of the outcome measures (ADAS13, CDR-SB, MMSE and amyloid PET centiloid) along the disease course. Based on longitudinal observations, subject samples were aligned to these mean trajectories by including latent-time variables that described the subject-level shifts in disease progression.

Let $y_{ijk}$ denote subject *i* ’s observation of the *k* th outcome measure at timepoint *j* ($t_{ij}$ years after the baseline visit). The mean trajectory $\theta_{k}$ of each outcome over the disease continuum was estimated from the model


(2) $$y_{ijk}=\theta_{k}(t_{ij}+s_{\mathrm{fixed}(i)}+s_{i})+x_{ik}+e_{ijk},$$

where we will refer to the time argument $t_{ij}+s_{\mathrm{fixed}(i)}+s_{i}$ that is shared across outcome measures as *disease time*.

The fixed effects of the model are $\theta_{k}$ and $s_{\mathrm{fixed}(i)}$, while $s_{i}$ and $x_{ik}$ are subject-level random effects and $e_{ijk}$ describes the residual variation. The latter three terms are assumed to be normally distributed. The parameters were modelled as follows:

-
$\theta_{k}$ : natural cubic spline with 9 degrees of freedom (DoF).
-
$s_{\mathrm{fixed}(i)}$ : fixed effect time-shift describing the average shift in disease time subject *i*’s baseline diagnostic group (i.e. CU-Aβ^−^, CU-Aβ^+^, MCI-Aβ^+^ and ADD-Aβ^+^) and baseline age.
-
$s_{i}$ : random effect time-shift describing the time deviation of subject *i* relative to their baseline group and age.
-
$x_{ik}$ : random effect intercept describing subject *i*’s consistent deviation in outcome measure *k*, an unstructured covariance matrix was used to model the correlation across outcomes.
-
$e_{ijk}$ : independent identically distributed Gaussian noise with separate variance parameters for each outcome *k*.

Predicted disease time ${\tilde{t}}_{ij}=t_{ij}+{\hat{s}}_{\mathrm{fixed}(i)}+{\hat{s}}_{i}$ for subject *i* was computed by inserting the maximum likelihood estimate of the baseline status fixed effect for subject ${\hat{s}}_{\mathrm{bl}\;\mathrm{status}(i)}$ and the maximum *a posteriori* prediction of the random shift ${\hat{s}}_{i}$ under the maximum likelihood estimates.

Time 0 on the estimated disease continuum was shifted to represent the time at which amyloid pathology exceeds normal bounds. Within the context of the model, it was defined as the time at which median CSF Aβ_1–42_ exceeded the 95th percentile of the Healthy (Aβ^−^) group.

##### Predicting trajectories across ‘estimated disease time’

The estimated disease times for each subject were used to compare trajectories of hippocampal volume and the five texture principal components (PCs). Values of these outcome variables $v_{ij}$ for subject *i* at predicted disease time ${\tilde{t}}_{ij}$ were modelled by the following random-effects model


(3) $$v_{ij}=\theta ({\tilde{t}}_{ij})+x_{i}+\varepsilon_{ij},$$

where θ is a natural cubic spline, $x_{i}$ is a subject-level random intercept that is assumed to be zero-mean normal distributed with variance $\tau^{2}$ and $\varepsilon_{ij}$ is the zero-mean normally distributed residual error with variance $\sigma^{2}$. These models were fitted with between 3 and 6 DoF splines, and the best-fitting model for each outcome variable was selected using Bayesian Information Criterion (BIC) with maximum likelihood estimation. The selected model was re-fitted using restricted maximum likelihood estimation.

Individual trajectories are shown relative to the Healthy group, by normalizing trajectories against the CU-Aβ^−^ group median and range. Let $q_{0.5}$ and $q_{0.95}$ denote respectively the median and 95% quantiles (in direction of abnormality) of outcome values observed in patients that were classified as CU-Aβ^−^ at baseline. Outcome variable abnormality at disease time *t* relative to this group was computed as


(4) $$\frac{1}{q_{0.95}-q_{0.5}}(\hat{\theta }(t)-q_{0.5}),$$

where $\hat{\theta }$ denotes the restricted maximum likelihood estimate of θ. This scale shows the magnitude of variable change over time, but it does not provide information on the measurement error at any given time, in other words, how sensitive that variable is to measuring change in a given individual. Sensitivity to change of each outcome variable was computed from the linear mixed-effects model as the derivative of the estimated trajectory divided by the standard error of the residuals (having removed subject-level intercepts)


(5) $$\frac{1}{\hat{\sigma }}\frac{d}{dt}\hat{\theta }(t).$$

Trajectories for variables along these scales were predicted for the entire continuum of disease times, excluding extreme 5% quantiles in order to minimize spurious effects from areas with few data points on which to calculate the trajectory.

##### Predicting trajectories across age and disease time simultaneously

The single-timescale models described in the previous section do not fully disentangle the effect of increasing age and estimated disease time. In order to directly explore the effect of age on hippocampal texture and volume, we used dual-timescale models (described by Raket *et al*.^37^) where we simultaneously modelled outcomes as a function of both progressive age and the estimated disease time calculated previously.

For each of the six outcome variables (five texture components and hippocampal volume), five models for describing the relation to disease progression and age were considered, and the best was selected using BIC. In the models below, the notation for spline functions, random effect intercepts and residual errors are the same as in the previous sections.

The five models are all of the form


$$y_{ij}=\theta ({\tilde{t}}_{ij},a_{ij})+x_{i}+\varepsilon_{ij},$$

where *θ* describe the trajectory as a function of predicted disease time ${\tilde{t}}_{ij}$ and age $a_{ij}$, $x_{i}$ is a subject-level random intercept modelled as a zero-mean normal distribution and $\varepsilon_{ij}$ describes the independent normal residual error. The five models differ in their choice of trajectory *θ* :


(6) $$\mathrm{No}\;\mathrm{effect}\;\mathrm{of}\;\mathrm{disease}\;\mathrm{progression}\;\mathrm{or}\;\mathrm{age}:\;\theta ({\tilde{t}}_{ij},a_{ij})=k$$

(7) $$\mathrm{Only}\;\mathrm{effect}\;\mathrm{of}\;\mathrm{disease}\;\mathrm{progression}:\;\theta ({\tilde{t}}_{ij},a_{ij})=\theta_{d}({\tilde{t}}_{ij})$$

(8) $$\mathrm{Only}\;\mathrm{effect}\;\mathrm{of}\;\mathrm{progressive}\;\mathrm{age}:\;\theta ({\tilde{t}}_{ij},a_{ij})=\theta_{a}(a_{ij})$$

(9) $$\mathrm{Additive}\;\mathrm{effect}\;\mathrm{of}\;\mathrm{disease}\;\mathrm{progression}\;\mathrm{and}\;\mathrm{age}:\;\theta ({\tilde{t}}_{ij},a_{ij})=\theta d({\tilde{t}}_{ij})+\theta_{a}(a_{t0(i)})$$

Interaction effect of disease progression and age:


(10) $$\theta ({\tilde{t}}_{ij},a_{ij})=\theta_{d}\;\mathrm{int}({\tilde{t}}_{ij})\theta_{a}\;\mathrm{int}(a_{t0(i)})+\theta_{d}({\tilde{t}}_{ij})+\theta_{a}(a_{t0(i)}).$$

To avoid capturing progressive time twice, age at predicted disease Time 0 ($a_{t0(i)}$) is used in models where both time scales are present, while progressive age ($a_{ij}$) is used when age is the sole predictor. For each of these models, Schwartz BIC was used to select the most parsimonious model with between 1 and 6 DoF on each spline term.

#### Independent information in texture and volume: predicting cognitive decline

In order to investigate if hippocampal texture provides additional useful information on top of that provided by volume to predict cognitive decline, we compared the variance explained by three sets of three linear regression models predicting future cognitive ability (over 2 years) in people without a diagnosis of dementia at baseline (CU and MCI groups).


(11) CogScore∼CogScore_bl+Age_bl+Sex+Education+ICV

(12) CogScore∼CogScore_bl+Age_bl+Sex+Education+ICV+HV

(13) $$\begin{array}{rl} \mathrm{CogScore}∼ & \mathrm{CogScore}\_\mathrm{bl}+\mathrm{Age}\_\mathrm{bl}+\mathrm{Sex}+\mathrm{Education}\\ & +\mathrm{ICV}+\mathrm{HV}+Tx\;PC1+Tx\;PC2+Tx\;PC3\\ & +Tx\;PC4+Tx\;PC5, \end{array}$$

where CogScore is ADAS13, CDR-SB or MMSE and HV is hippocampal volume. Models were bootstrapped with 1000 repetitions in order to calculate confidence intervals and perform statistical comparison between models, using two-sample *t*-tests, adjusting for multiple comparisons across the nine models (three scores × three models) using the Bonferroni–Holm method (*rstatix* R package). These models were bootstrapped and adjusted *R*^2^ ($R_{\mathrm{adj}}^{2}$) values were compared across models using two-sample *t*-tests. Critically, $R_{\mathrm{adj}}^{2}$ (as opposed to *R*^2^) accounts for the varying DoF between models and penalizes models with a greater number of variables. All statistical tests were two-tailed, and an alpha of 0.05 was used to indicate statistical significance. All analyses were performed, and plots created in R v4.1.1.

## Results

### Data analysis procedure

To address our study aims, we leveraged both cross-sectional and longitudinal analysis techniques using data collected as part of the ADNI project. To determine whether hippocampal texture is sensitive to the earliest pathological changes in Alzheimer’s disease, we ran linear mixed-effects models on baseline data. Using these models, we determined whether any detectible differences existed in volume or texture across disease stages, including between CU people with and without evidence of amyloid pathology, one of the first known detectible changes in Alzheimer’s disease. To assess texture change across the Alzheimer’s disease continuum, we used a multivariate spline-based mixed-effects model to estimate a ‘predicted disease time’ for all participant timepoints based on amyloid PET and cognitive scores. Predicted disease times were used as a scale on which to compare trajectories of various biomarkers, including volume and texture of the hippocampus.

### Feature reduction

**Figure 2:**
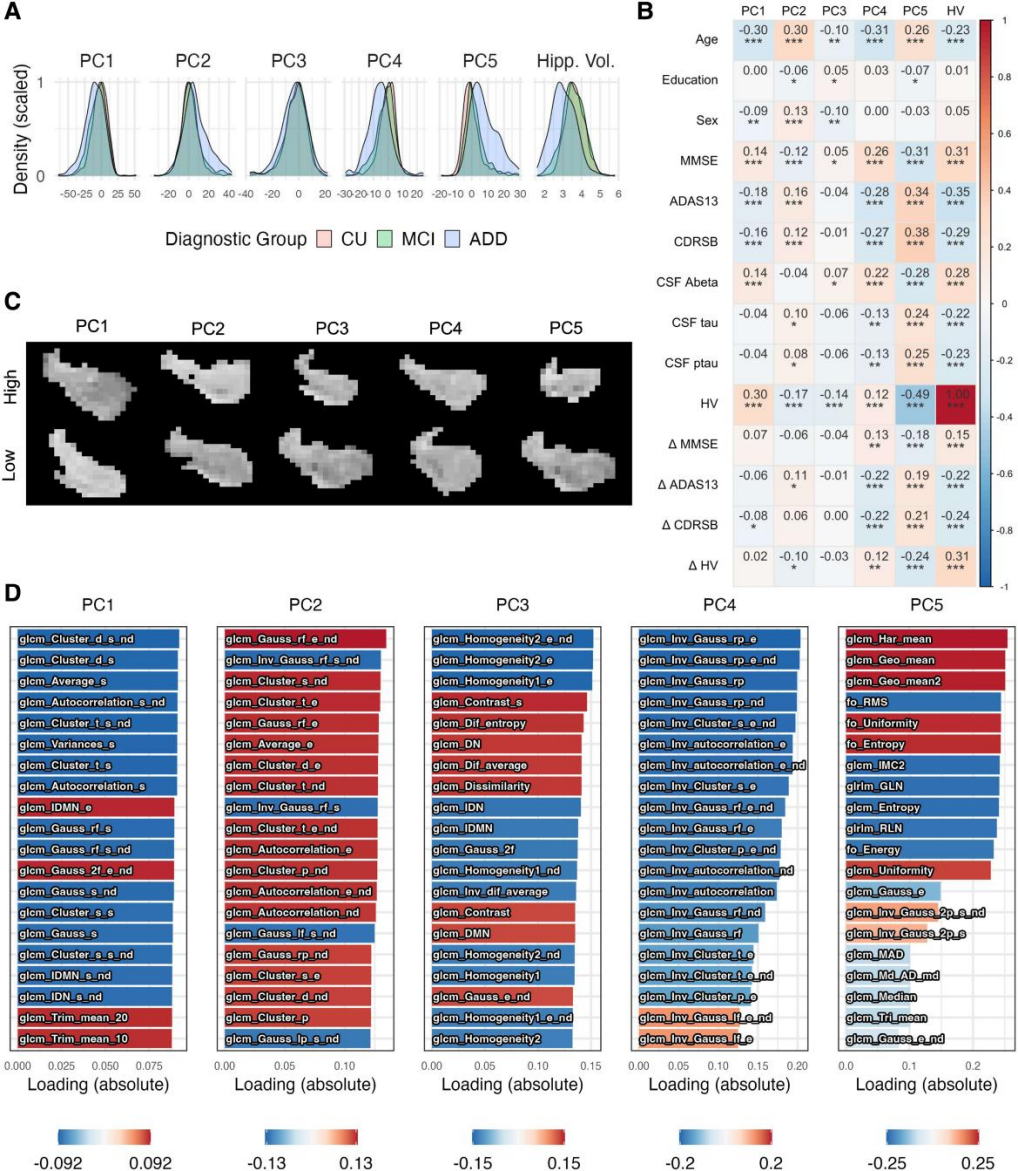
**Description of texture components.** (**A**) Probability density functions to show spread of each texture component, and volume, in each of the three clinical diagnostic groups. This figure shows all participants for whom texture analysis was run, including those without amyloid status data—the CU group is therefore not split into amyloid positivity subgroups. Units for volume are shown here in cm^3^. (**B**) Spearman partial correlation plot of the relationship between each texture component and volume, other variables of interest; Spearman partial *r*-values are shown for each comparison, with stars indicating significance levels: *P < 0.05, **P < 0.001, ***P < 0.0001. Partial correlations control for age, education, sex and intracranial volume. Change-over-time variables (Δ) are calculated as difference scores over 2 years. (**C**) Examples of hippocampi with extreme high/low values for each texture component. Each image shows a coronal view of left hippocampal ROI from an amyloid-negative CU participant. Some differences in intensity or clustering are evident, but overall, systematic differences between components are unclear upon visual inspection alone. More examples are shown in Supplementary Fig. 2. (**D**) Loading of top 20 texture variables onto each PC. Absolute values are shown on the *x*-axes, with direction of loading indicated by colour (red: positive, blue: negative). Variable names are prefixed with either ‘fo’ (first-order statistics), ‘glcm’ or ‘glrlm’. A full list of loadings is available in Supplementary Fig. 1 and Supplementary Table 1. ADAS13, Alzheimer’s Disease Assessment Scale (13 questions); ADD, Alzheimer’s disease dementia; CDR-SB, Clinical Dementia Rating scale Sum of Boxes; CU, cognitively unimpaired; HV, hippocampal volume; MCI, mild cognitive impairment; MMSE, Mini Mental State Examination; PC, principal component.

Five principal components of texture explained ≥5% of the variance across CU participants, respectively describing 43, 19, 14, 7, 5% of the variance in CU-Aβ^−^ hippocampal texture (total: 88%). Probability density functions in Fig. 2A show the distribution shapes of each of these components across diagnostic groups before correcting for covariates such as age, sex and education. Correlations with variables of interest and visual appearance of each component are shown in Fig. 2B and C, respectively. Top variable loadings on each PC are shown in Fig. 2D, with full variable loadings shown in Supplementary Fig. 1 and Supplementary Table 1.

### Differences in texture and volume between groups at baseline

**Figure 3:**
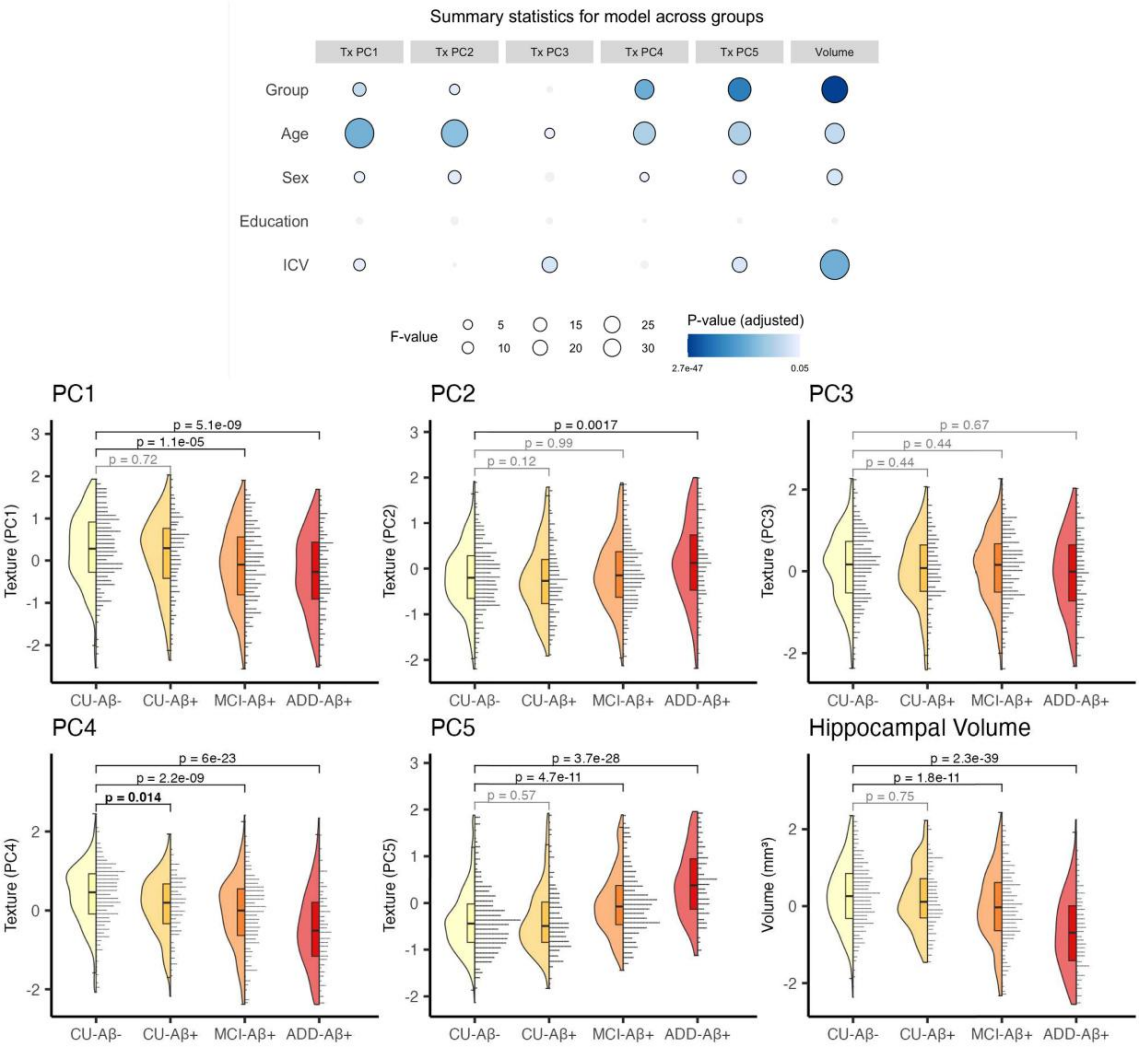
**Group differences in hippocampal texture and volume at baseline.** Top: summary statistics from linear mixed-effects model showing the strength of the association between each predictor/covariate and each texture component/volume. The size of circles represents the *F*-statistic of each predictor, while the shade represents the false discovery rate-adjusted *P*-value. Non-significant effects are shown in grey. An overall effect of group was seen in all variables except Tx PC3, with the strongest effects seen for volume, Tx PC5 and Tx PC4. Strong effects of age were also evident for each variable though this effect was considerably weaker for Tx PC3. *P*-values were false discovery rate-adjusted across all tests for multiple comparisons. Bottom: groupwise raincloud plots showing data for each texture component and volume. *P*-values shown are that of the main effect of group in pairwise models. Non-significant effects are shown with grey brackets. Tx PC4 was the only variable where a difference between CU-Aβ^−^ and CU-Aβ^+^ groups is seen. The *P*-values shown are adjusted for false discovery rate correction. ADD, Alzheimer’s disease dementia; CU, cognitively unimpaired; ICV, intracranial volume; MCI, mild cognitive impairment; PC, principal component

We observed significant differences in certain texture components (Tx PC) between diagnostic groups (Fig. 3). Early in the Alzheimer’s disease continuum, differences in texture component 4 (Tx PC4) were observed between the CU-Aβ^−^ and CU-Aβ^+^ groups. Differences in Tx PC1, Tx PC5 and hippocampal volume were detectible at the stage of MCI. Differences in Tx PC2 were detected in people with dementia. Tx PC3 was not significantly different from the Healthy group at any stage. Statistics for these tests are presented below.

Linear mixed-effects models on hippocampal texture at baseline revealed significant differences between diagnostic groups in Tx PC1 [*F*(3,1100) = 14.8, *f* = 0.20, *P*_adj_ < 0.0001], Tx PC2 [*F*(3,1055) = 7.04, *f* = 0.14, *P*_adj_ < 0.001], Tx PC4 [*F*(3,1077) = 39.0, *f* = 0.33, *P*_adj_ < 0.0001], Tx PC5 [*F*(3,1032) = 60.0, *f* = 0.42, *P*_adj_ < 0.0001] and volume [*F*(3,1107) = 80.8, *f* = 0.47, *P*_adj_ < 0.0001], but not Tx PC3 [*F*(3, 1050) = 0.973, *f* = 0.05, *P*_adj_ = 0.467]. We also observed consistent effects of age across all texture components and volume (*F*’s = 6.24–103, *P*’s < 0.020).

In order to explore when texture changes occurred in the disease course, we compared the CU-Aβ^−^ group to every other group by re-running models (1) with only two groups at a time.

#### Differences between CU-Aβ^−^ and -Aβ^+^ groups

We observed a significant difference between CU-Aβ^−^ and CU-Aβ^+^ groups only for Tx PC4 [*F*(1,564) = 7.21, *d* = 0.23, *P*_adj_ = 0.014].

#### Differences between Cu-Aβ^−^ and MCI-Aβ^+^ groups

We found a significant difference between CU-Aβ^−^ and MCI-Aβ^+^ groups in Tx PC1 [*F*(1,710) = 21.2, *d* = 0.35, *P*_adj_ < 0.0001], Tx PC4 [*F*(1,695) = 39.0, *d* = 0.47, *P*_adj_ < 0.0001], Tx PC5 [*F*(1,685) = 47.4, *d* = 0.53, *P*_adj_ < 0.0001] and volume [*F*(1,715) = 49.9, *d* = 0.53, *P*_adj_ < 0.0001].

#### Differences between Cu-Aβ^−^ and ADD-Aβ^+^ groups

Finally, differences between the CU-Aβ^−^ and ADD-Aβ^+^ groups were observed for Tx PC1 [*F*(1,564) = 37.2, *d* = 0.51, *P*_adj_ < 0.0001], Tx PC2 [*F*(1,492) = 11.2, *d* = 0.30, *P*_adj_ = 0.002], Tx PC4 [*F*(1,547) = 111, *d* = 0.90, *P*_adj_ < 0.0001], Tx PC5 [*F*(1,519) = 142, *d* = 1.05, *P*_adj_ < 0.0001] and volume [*F*(1,565) = 210, *d* = 1.22, *P*_adj_ < 0.0001].

No significant pairwise group differences were found for Tx PC3. Comparisons between all other groups are shown in supplemental information (Supplementary Fig. 3; Supplementary Table 2).

### Longitudinal analysis: texture trajectories

#### Calculating disease time

**Figure 4:**
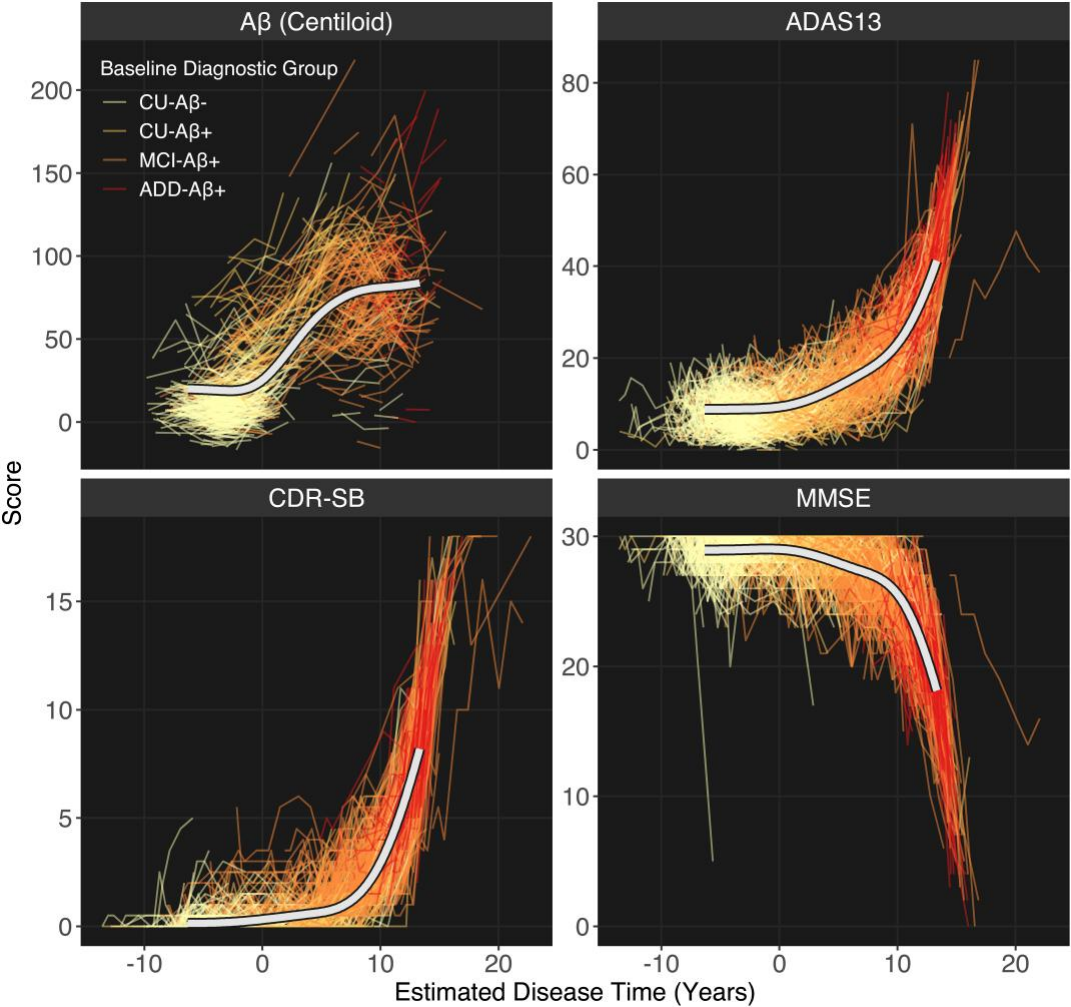
**Trajectories of staging variables for longitudinal modelling in the present sample.** The *x*-axis shows the estimated disease time, upon which all variables can be staged. Disease onset occurs at Time 0. Each individual used for staging with multiple timepoints is shown as a coloured line. The estimated mean trajectory is modelled for each variable and displayed on top (white line). ADAS13, Alzheimer’s Disease Assessment Scale (13 questions); ADD, Alzheimer’s disease dementia; CDR-SB, Clinical Dementia Rating scale Sum of Boxes; CU, cognitively unimpaired; MCI, mild cognitive impairment; MMSE, Mini Mental State Examination.

To assess texture change across the Alzheimer’s disease continuum, we used the previously described multivariate disease progression model to estimate a latent ‘disease time’ variable for all participant timepoints. The model was trained on a total of 6465 sessions of data over 1416 participants. Of these, 441 were in the CU-Aβ^−^ group, 231 CU-Aβ^+^, 480 MCI-Aβ^+^ and 264 ADD-Aβ^+^. Overall, disease time was estimated for a total of 9068 timepoints. After fitting, the data spanned an estimated disease course of 236 months (19.7 years). Figure 4 shows the predicted staging of individuals across the four variables used for staging.

#### Fitting longitudinal trajectories of hippocampal texture and volume

The estimated disease time calculated above (2) was used as a continuous scale on which to stage other variables (3). After exclusion of 5% extreme quantiles, these trajectories were calculated using 8164 timepoints over a subset of 1403 participants of the 1416 participants used above. Longitudinal spline trajectories of all five PC texture components were best fitted by 3 DoF models (as defined by BIC), while the trajectory of hippocampal volume was best fitted by a 5 DoF model.

**Figure 5:**
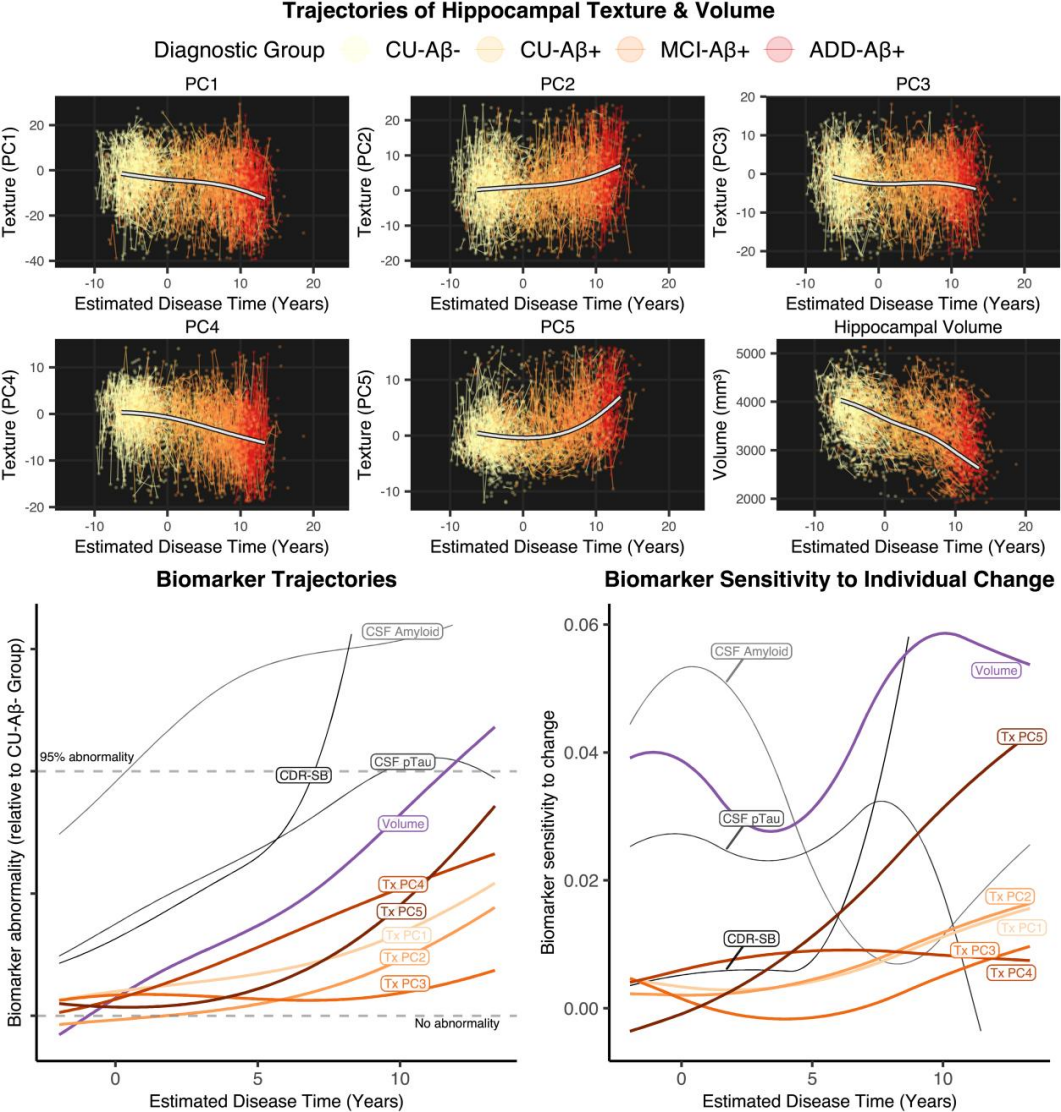
**Estimated trajectories of disease marker variables.** Top: individual participant trajectories for each hippocampal texture component and volume, with overlaid spline curves. Bottom: spline curves across the calculated disease continuum for each hippocampal texture component (red hues), hippocampal volume (purple) and other variables, including CDR-SB. CSF amyloid and CSF pTau (grey hues). Left plot shows splines normalized relative to the Healthy group. Dashed lines represent levels of no abnormality (Healthy group median) and 95% abnormality (95th percentile of Healthy group). Texture PC4 appeared to change earlier than other texture components, with the greatest overall texture change seen in PC5. After Time 0, volume remained more abnormal than all texture components, relative to the CU-Aβ^−^ group. Right plot shows each variable’s sensitivity to change at the participant level. Sensitivity to change of each biomarker was computed from the linear mixed-effects model as the derivative of the estimated biomarker trajectory divided by the residual standard deviation texture components retain relatively low sensitivity to change across the disease course, except texture PC5 which does increase at later stages, though not above the level of hippocampal volume. ADD, Alzheimer’s disease dementia; CDR-SB, Clinical Dementia Rating scale Sum of Boxes; CU, cognitively unimpaired; MCI, mild cognitive impairment; PC, principal component; Tx, texture.

Figure 5 shows trajectories, relative to the CU-Aβ^−^ group, of hippocampal texture and volume alongside CSF biomarker trajectories (Aβ and pTau) as well as a representative cognitive trajectory (CDR-SB). Hippocampal volume always appeared more abnormal than texture relative to the CU-Aβ^−^ group after Time 0.

Plotting sensitivity to change revealed limited sensitivity to change in texture measures compared with other biomarker variables. In other words, individual-level change is difficult to measure accurately, especially at early disease stages, despite evidence for early texture changes on a groupwise basis. On this plot, we also see that CSF Aβ was the most sensitive marker of change at early stages of disease. At around 5 years, CSF pTau and hippocampal volume increase in their sensitivity, with volume appearing more sensitive than any other measure. After ∼8 years, cognition (CDR-SB) becomes the most sensitive marker to change and remains so for the remainder of the disease course.

Variable trajectories were plotted across a total 184-month (15.3-years, from −2 to 13.3 years) time period. We avoided estimating trajectories at the very earliest timepoints as more than 2 years before Time 0, staging accuracy may be lower as the variability in the four staging variables (amyloid, ADAS, CDR-SB and MMSE) is more likely due to other factors than disease severity, such as noise.

#### Dissociating effects of age and disease

**Figure 6:**
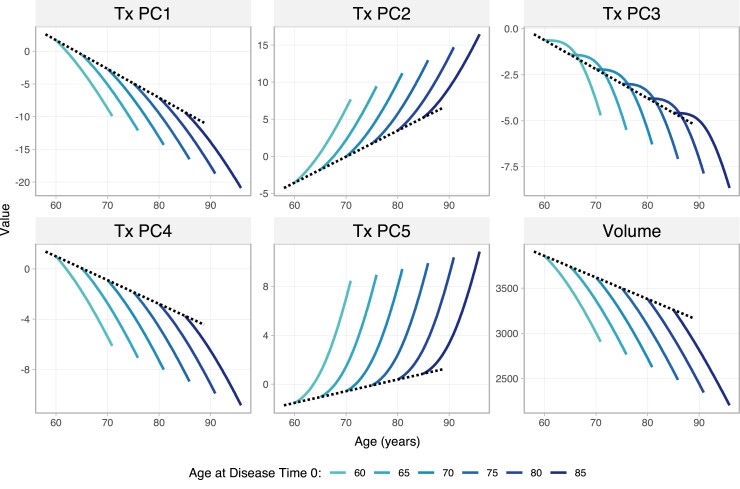
**Interactions between age and longitudinal change in hippocampal texture and volume.** Texture change was best described by additive models of disease time and age, indicating independent effects of both for all components. Volume change was best described by an interaction term between disease time and age, indicating faster atrophy in people who have later-onset disease. The black dotted line represents the age trajectory before onset of Alzheimer’s disease pathology (Time 0) and is therefore analogous to a ‘healthy ageing’ trajectory. Each blue curve represents a 15-year pathological trajectory for people with different age of disease onset. PC, principal component; Tx, texture.

To assess how texture changes with age, and how that interacts with change due to disease, we tested whether dual-timescale models incorporating both age and predicted disease time [as either additive (equation 9) or interaction (equation 10) terms] would have better fit than single-timescale models (equations 7 and 8) or a null model (equation 6). Texture component trajectories were all best explained by an additive model of age and disease time (as determined by BIC). That is, texture changes independently along both scales. Age effects were best fit with a single DoF, indicating approximately linear change with age (Fig. 6). The disease time term for Tx PC1, Tx PC2, Tx PC4 and volume was best fitted as a spline with 2 DoF, whereas those for Tx PC3 and Tx PC5 were best fitted with a 3 DoF spline model.

Volume was the best fit by a model with an interaction term for age and disease time (BIC = 54 801). That is, the disease trajectory differed depending on the age of disease onset. The model suggested that volume change due to disease was faster for people with a later disease onset, and slower for those whose disease started earlier in life. Figure 6 shows the trajectories of each texture variable and volume.

### Texture provides additional information to volume

**Figure 7:**
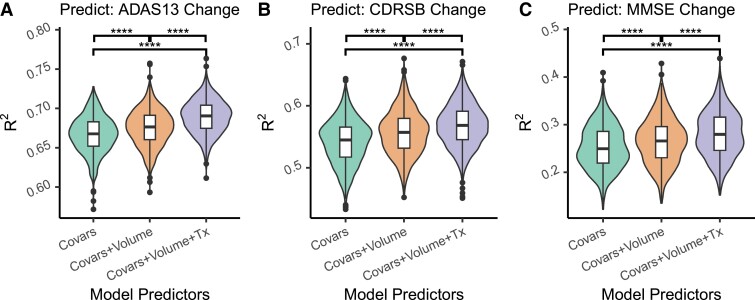
**Variance explained ($R_{\mathrm{adj}}^{2}$) for linear models predicting cognitive decline.** Cognitive decline models predict cognitive scores (**A**: ADAS-13, **B**: CDR-SB, **C**: MMSE) 24 months after baseline of people without a diagnosis of dementia at baseline, with baseline cognitive score, baseline age, years of education, sex and intracranial volume as predictors. Adding volume to these models (orange/middle) significantly increased $R_{\mathrm{adj}}^{2}$. Adding texture (purple/right) significantly increased $R_{\mathrm{adj}}^{2}$ even further in all cases. Comparisons are made using 1000-bootstraped resamples of the data. Adjusted *P*-values, corrected across all tests, are shown. ****P_adj_ < 0.0001. ADAS13, Alzheimer’s Disease Assessment Scale (13 questions); CDR-SB, Clinical Dementia Rating scale Sum of Boxes; MMSE, Mini Mental State Examination.

In order to investigate if hippocampal texture provided additional useful information on top of that provided by volume alone, we ran three linear models [equations (11)–(13)] predicting cognitive decline over a 2-year period in CU and MCI groups (i.e. those without a diagnosis of dementia at baseline): using only covariates (baseline cognition, baseline age, sex, years of education, and intracranial volume), with hippocampal volume, and with the subsequent addition of all texture features (Fig. 7). Adding hippocampal volume to the model significantly increased $R_{\mathrm{adj}}^{2}$ in predicting cognitive decline of all three cognitive tests [ADAS13: *t*(1998) = 8.12, *d* = 0.36, *P*_adj_ < 0.0001; CDR-SB: *t*(1998) = 9.43, *d* = 0.42, *P*_adj_ < 0.0001; MMSE: *t*(1994) = 6.13, *d* = 0.27, *P*_adj_ < 0.0001]. The subsequent addition of texture to the models further increased $R_{\mathrm{adj}}^{2}$ in all cases [ADAS13: *t*(1986) = 14.1, *d* = 0.63, *P*_adj_ < 0.0001; CDR-SB: *t*(1993) = 7.49, *d* = 0.34, *P*_adj_ < 0.0001; MMSE: *t*(1999) = 7.05, *d* = 0.32, *P*_adj_ < 0.0001].

## Discussion

In this article, we quantified and examined hippocampal texture, a potential proxy for microstructural pathological change, across the Alzheimer’s disease continuum. We identified a significant difference in a single component of texture at the earliest stage of Alzheimer’s disease, between CU older adults with and without evidence of Aβ pathology. Differences in additional components of texture, and hippocampal volume, emerged later in the disease continuum along with the onset of cognitive impairment. Using a longitudinal modelling framework, we also show that while most elements of texture changed significantly over the course of the disease, these measures had low sensitivity for tracking individual textural change over time. Critically however, we show that texture provided additional information than was provided by volume alone, to more accurately predict future cognitive change.

### Texture is sensitive to presymptomatic pathology

Our cross-sectional analyses revealed a temporal ordering in which markers of brain structure become abnormal relative to healthy ageing. Texture PC4 was different at the CU-Aβ^+^ stage of Alzheimer’s disease—that is, CU older adults with amyloid biomarkers. This group is at significantly higher chance of future progression to Alzheimer’s disease than the CU-Aβ^−^ group.^38^ This component therefore appears to be sensitive to the very earliest stages of pathology and may represent a chance to detect damage to extant tissue in Alzheimer’s disease before the onset of cognitive impairment. We find that hippocampal volume is only detectibly different later, after the onset of cognitive impairment. Volume and other later-changing texture variables most likely reflect significant damage to the hippocampus, given the temporal colocalization with clinical presentation of cognitive impairment.

Our results support and build upon previous findings that a texture analysis approach can distinguish between people with MCI with and without amyloid biomarkers.^14,39^ Similarly, past research has shown differences in T_2_ signal heterogeneity, as opposed to mean signal, across the hippocampus in people with MCI compared with healthy older controls.^40^ Three of our five texture variables were able to detect a difference at this stage. However, to our knowledge, a difference in hippocampal texture in an asymptomatic group with known biomarkers for Alzheimer’s disease has not previously been reported. While this effect is relatively small (*d* = 0.23), our result provides robust evidence of the ability of texture to detect brain changes at very early stages of the Alzheimer’s disease continuum.

The question remains as to whether there is any direct biological analogue of PC4 (or any other component of texture). The factors that load strongly onto PC4 (e.g. negative loading of inverse right-polar Gaussian and inverse autocorrelation grey-level co-occurrence matrix factors) indicate that a ‘high-PC4’ hippocampus possesses a higher proportion of high-intensity voxels appearing adjacent to other high-intensity voxels. In other words, these cases display quantifiable clustering of hyperintense areas. This may indicate localized increases in CSF due to small amounts of atrophy or accumulation of paramagnetic materials. Future studies could characterize how microstructural changes, for example, neuroinflammation or blood vessel damage, affect conceptually interpretable changes in texture features. We have included speculative descriptions on the meaning of each texture feature in supplemental information.

An alternative interpretation of our results is that changes in texture features are driven by more macroscale changes across the hippocampus, such as different proportions of subfields, which have been shown to degrade at different rates due to Alzheimer’s disease pathology.^41^ A mixture of both models may be driving such textural changes. In support of the model that microstructural changes drive texture, SNIPE scores are a related measure that use non-local patch-based methods to segment^42^ and grade^43^ hippocampus. SNIPE scores depend on the similarity of patch intensity, contrast, as well as texture. In this approach, healthy adults and patients with Alzheimer’s disease can be differentiated using the SNIPE grading score.^43^ SNIPE looks at small patches (e.g. 7 × 7 × 7 voxels) of MR intensities and compares them to a pre-labelled library of MRIs of participants that are either healthy or patients with Alzheimer’s disease. The SNIPE score is a weighted average of the neighbourhood patch similarities in the training library. The patch-based method means that changes are more likely to be due to microstructural changes across a given patch that macroscale changes across the entire structure. These SNIPE scores can predict which healthy aging community-dwelling participants will progress to Alzheimer’s disease over a 12 years of follow-up period^15^ and differentiate patients that have stable MCI from those that will progress to Alzheimer’s disease.^16,17^

### Texture develops over the disease continuum and improves prediction of cognitive change

We used a multivariate modelling technique, adapted from previous work,^35,36^ to determine trajectories of change across the Alzheimer’s disease continuum. Cognitive tests provide accurate staging at the later stages of disease but do a poor job at earlier stages, due to limited variability. Amyloid PET, one of the earliest indicators of Alzheimer’s disease pathology detectible *in vivo* improved staging at the earliest points in the disease course. Overall, an almost 20-year disease timeline was estimated.

In line with our cross-sectional analyses, change in texture over the disease course is evident; however, hippocampal volume appears to change at a faster rate and, ultimately, to a greater degree than any texture component. Assessing the sensitivity of each variable to change on an individual level reveals that long-term texture trajectories are mainly driven by groupwise differences, and that individual change in texture is more difficult to measure accurately than volume change, likely attributable to noise factors across timepoints. We attempted to reduce this noise by averaging data across hemispheres; however, this is still an area for future improvement.

Even so, we demonstrate that texture features improve prediction of cognitive change in preclinical populations above and beyond the improvement provided by volume alone. This supports previous work showing that texture provides more information than volume in identifying Alzheimer’s disease,^15^ and classifying MCI patients who convert to Alzheimer’s disease from those who remain stable.^7,16,17^ We also support a finding that even after decorrelating texture and volume, texture was able to significantly predict cognitive decline in people with MCI over 24 months.^5^ Our findings validate texture as an important addition to existing clinical assessments alongside volumetry.

### Texture is a versatile tool for assessing tissue health

We also show dissociable effects of age and disease time in all texture components, as well as volume. This supports the use of texture as a generalizable measure of brain health in aging, as well as marker of Alzheimer’s disease pathology. Indeed, our measured texture features are likely sensitive to a myriad of microstructural changes that can affect T_1_ signal, such as water content, inflammatory markers or demyelination.^44,45^ It has been shown to be useful in characterization and detection of pathology due to: Parkinson’s Disease,^46^ tumours,^9^ and epilepsy.^47^ It is a strength of texture analysis that it is sensitive to this broad array of changes but given the variety of factors that can affect T_1_ signal, specificity remains a challenge. Specificity could be provided by distribution patterns of texture abnormality across the brain, in a similar fashion to cortical thickness or volume as measures of atrophy that are agnostic to underlying causes. Although textural changes in the context of preclinical Alzheimer’s disease have been previously shown to be limited to the medial temporal lobes,^7^ more detailed, targeted analyses remain to be conducted. For example, the utility of texture as a method of assessing structural covariance, and multi-regional patterns of disease-related changes that covary over time.

### Conclusions and future directions

With further validation, texture analysis of hippocampal MRI could be used in conjunction with other measures in initial screening of clinical trial cohorts for individuals who are at risk for Alzheimer’s disease, prior to more expensive and invasive tests that provide higher specificity. Given that MRI scans are often conducted to assess macrostructural atrophy (as measured by volume), or to rule out other causes of disease symptoms, texture analysis can feasibly be added to existing clinical pipelines to provide additional information on microstructural tissue quality.

We have deliberately explored texture on standard 1 mm isotropic T_1_-weighted MRI scans, given their wide clinical accessibility, in order to maximize the clinical relevance of our findings. However, a consensus on methodological approaches and improvements to reduce timepoint-to-timepoint noise are necessary if texture is to become a marker of individual change over time. Texture analysis of other MRI modalities may also hold potential for detecting microstructural abnormalities such as quantitative T_1_ and T_2_, magnetization transfer or quantitative susceptibility mapping.

## Supplementary Material

fcad195_Supplementary_Data

## Data Availability

Data used in the preparation of this article were obtained from the Alzheimer’s Disease Neuroimaging Initiative (ADNI) database (adni.loni.usc.edu). All analyses were performed using openly available code packages. Custom wrapper scripts for measuring texture are available on *github* at https://github.com/Alfiew/Texture_processing_ADNI. Analysis scripts are available at https://github.com/Alfiew/Longtiudinal_trajectories_of_hippocampal_texture.
