# Supplementary material for: Longitudinal changes in hippocampal texture from healthy aging to Alzheimer’s disease
Source: Brain Commun. 2023 Jul 5;5(4):fcad195. doi: 10.1093/braincomms/fcad195 (PMC10351670; doi:10.1093/braincomms/fcad195)
Supplement: fcad195_Supplementary_Data [file fcad195_supplementary_data.pdf]

Supplemental Figure 1 | Full list of texture variable PC loadings. Absolute values are shown on the x−axes, with direction of loading indicated by color (red: positive, blue: negative). Variable names are prefixed with either ‘fo‘ (first order statistics), ‘glcm‘ (grey−level co−occurance matrix) or ‘glrlm‘ (grey−level run−length matrix).

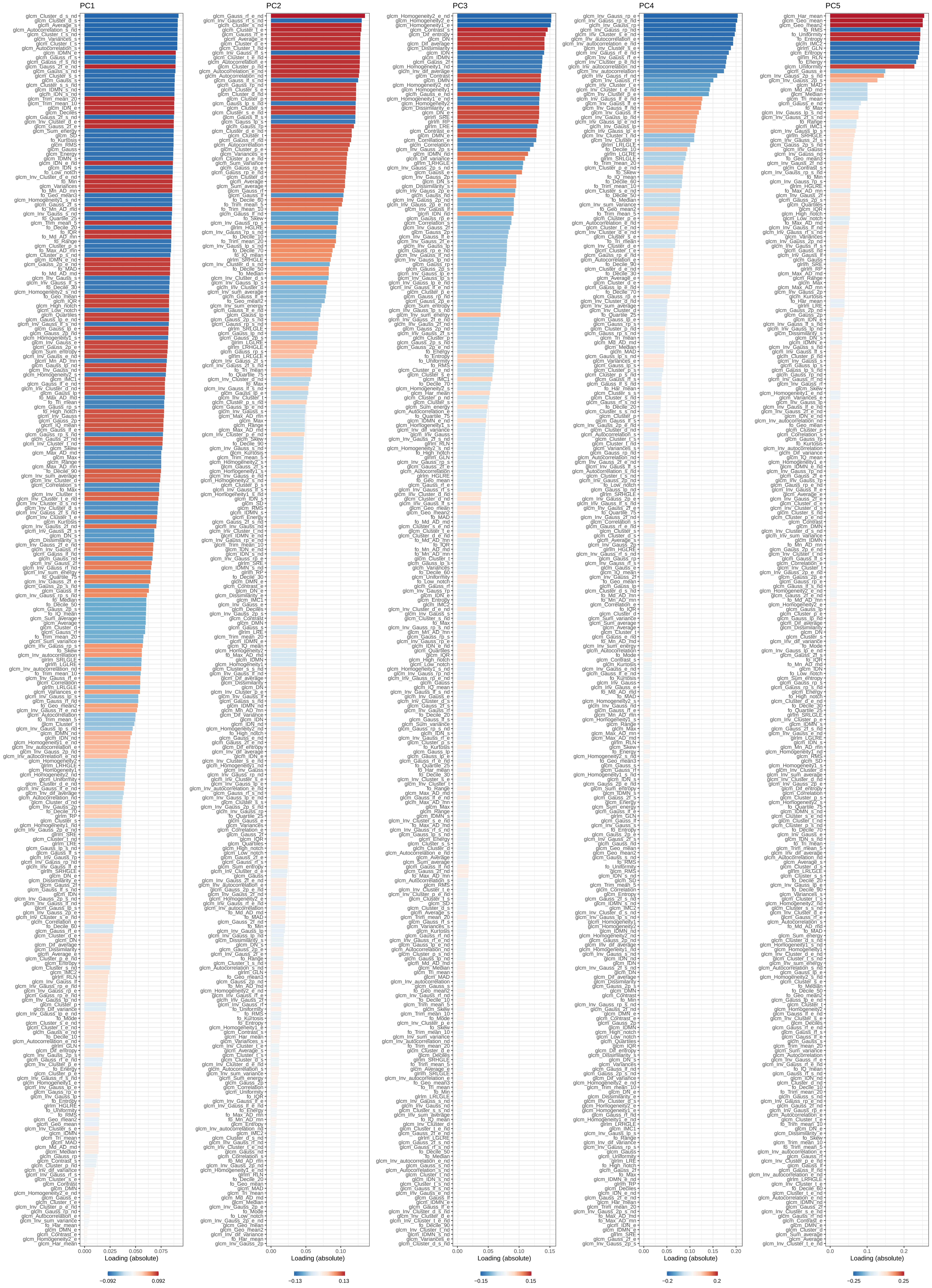

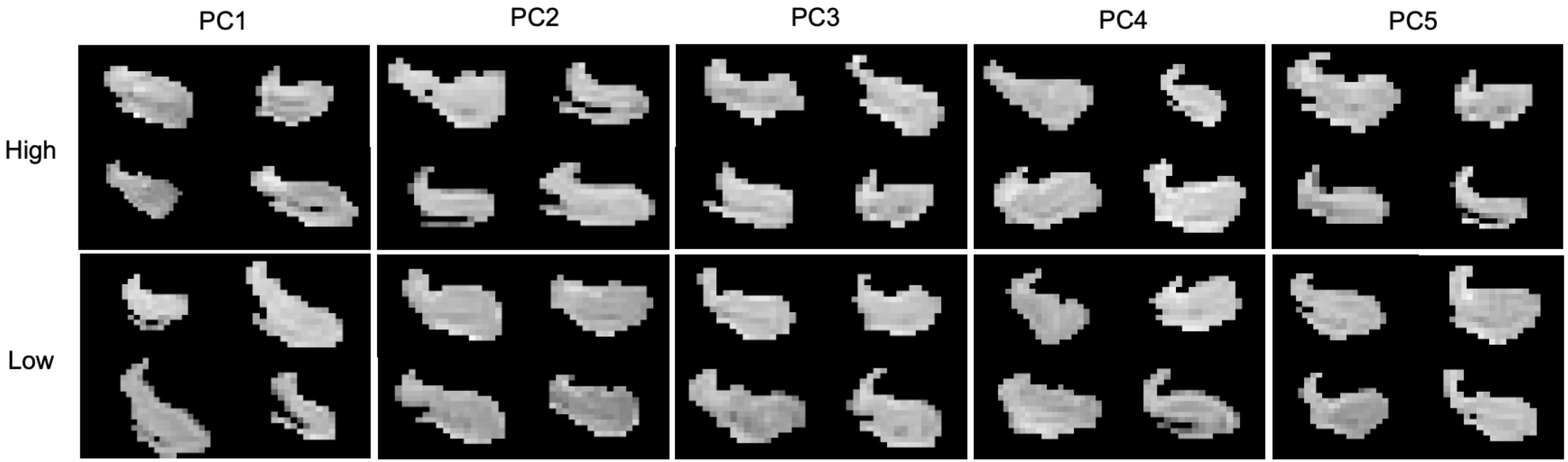

Supplementary Figure 2 | ***Example hippocampi with extreme high and low values for each texture component.*** *Each image shows a coronal view of left hippocampal ROI from a cognitively unimpaired participant. Some differences in intensity or clustering are evident, but overall, systematic differences between components are unclear upon visual inspection alone.*

Supplemental Results

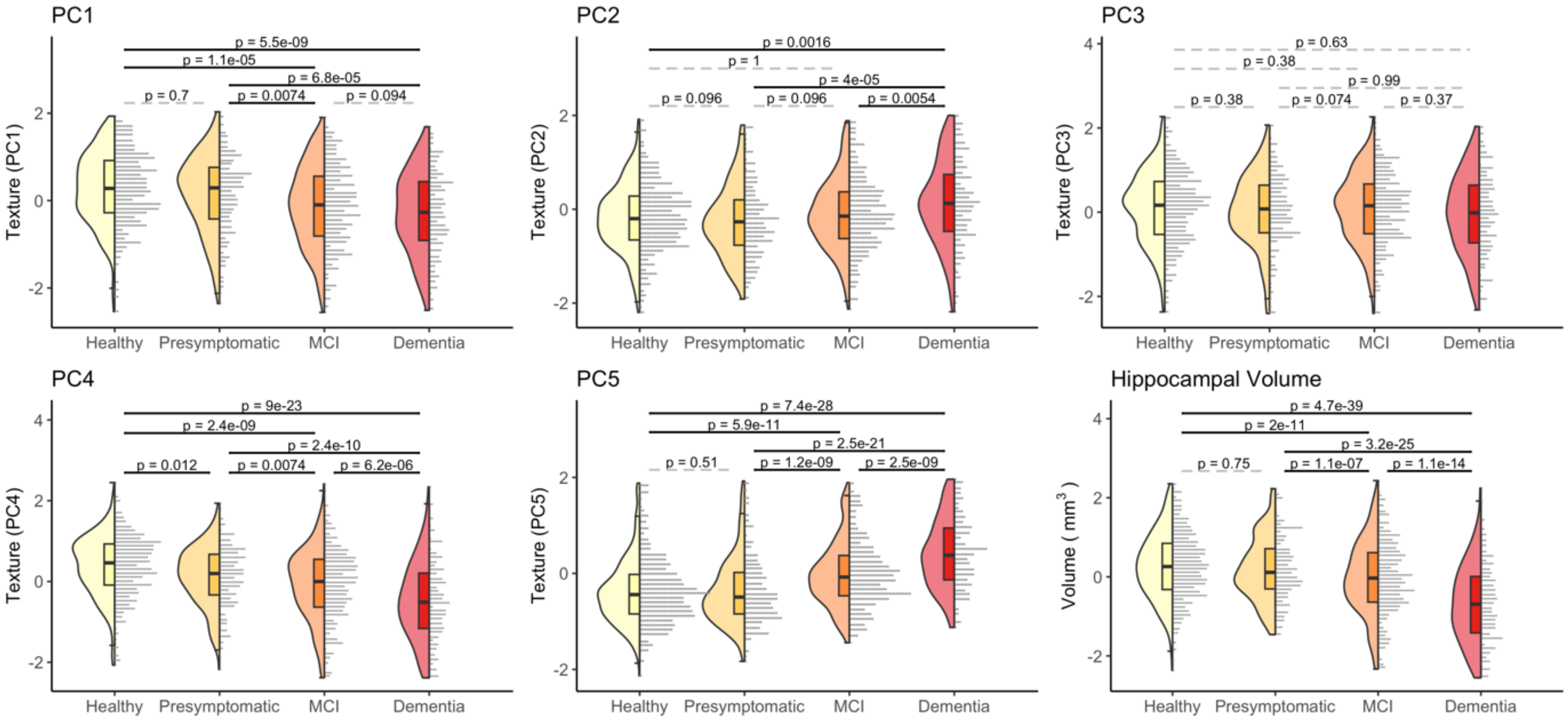

Supplemental Figure 3 | ***Groupwise raincloud plots showing data for each texture component and volume****. P-values shown are that of the main effect of group in pairwise models. These P-values are FDR-adjusted for multiple comparisons. Full statistics for each of these models can be seen in Supplemental Table 2.*

Supplementary Table 1 | List of texture variables included in Principal Component Analysis, with loadings on each component. Key abbreviations: fo = First order statistics; glcm = Grey level co-occurance matrix; glrlm = gray-level run-length matrix. A full description of all variables is provided by Kolossváry et al. (2017).

**Texture Variable**	**PC1**	**PC2**	**PC3**	**PC4**	**PC5**
fo_Median	-0.0605	0.0827	-0.0051	-0.0787	-0.0084
fo_Mode	0.0206	-0.0023	0.0109	-0.0180	0.0162
fo_Geo_mean	0.0829	0.0027	-0.0403	0.0216	0.0244
fo_Geo_mean2	0.0522	-0.0769	0.0124	0.0772	-0.0082
fo_Geo_mean3	-0.0858	0.0166	0.0090	0.0123	-0.0581
fo_Har_mean	-0.0036	0.0006	-0.0212	-0.0383	-0.0387
fo_Trim_mean_5	-0.0496	0.1017	-0.0092	-0.0765	-0.0028
fo_Trim_mean_10	-0.0549	0.0968	-0.0100	-0.0823	-0.0034
fo_Trim_mean_20	-0.0577	0.0930	-0.0096	-0.0863	-0.0049
fo_IQ_mean	-0.0601	0.0871	-0.0073	-0.0840	-0.0058
fo_Tri_mean	-0.0780	0.0586	0.0090	-0.0681	-0.0123
fo_Mn_AD_mn	0.0859	0.0079	-0.0341	0.0203	0.0207
fo_Mn_AD_md	0.0857	0.0163	-0.0346	0.0187	0.0153
fo_Md_AD_mn	0.0850	0.0044	-0.0356	0.0205	0.0178
fo_Md_AD_md	0.0837	0.0207	-0.0370	0.0156	0.0100
fo_MAD	0.0837	0.0207	-0.0370	0.0156	0.0100
fo_Max_AD_mn	-0.0846	-0.0081	0.0178	0.0006	-0.0549
fo_Max_AD_md	-0.0785	-0.0364	0.0198	0.0007	-0.0510
fo_RMS	0.0147	-0.0147	-0.0590	0.0095	-0.2440
fo_Min	0.0861	-0.0204	-0.0085	-0.0074	0.0560
fo_Max	-0.0730	-0.0545	0.0302	-0.0018	-0.0780
fo_IQR	0.0850	0.0090	-0.0356	0.0198	0.0153
fo_Low_notch	-0.0864	0.0022	0.0318	-0.0292	-0.0153
fo_High_notch	0.0779	0.0339	-0.0426	-0.0024	0.0147
fo_Range	-0.0849	-0.0176	0.0205	0.0031	-0.0713
fo_Skew	0.0560	-0.0961	0.0100	0.0845	0.0033
fo_Kurtosis	-0.0872	-0.0142	0.0226	-0.0159	-0.0241
fo_Energy	0.0171	-0.0084	-0.0596	0.0136	-0.2326
fo_Uniformity	-0.0147	0.0147	0.0590	-0.0094	0.2440
fo_Entropy	-0.0149	0.0142	0.0591	-0.0098	0.2436
fo_Quartile_25	-0.0853	0.0280	0.0215	-0.0495	-0.0145
fo_Quartile_75	0.0645	0.0583	-0.0469	-0.0261	0.0129
fo_Decile_10	0.0192	-0.0931	0.0122	0.0943	-0.0052
fo_Decile_20	-0.0852	0.0031	0.0246	-0.0364	-0.0109
fo_Decile_30	-0.0830	0.0398	0.0211	-0.0600	-0.0145
fo_Decile_50	-0.0605	0.0827	-0.0051	-0.0787	-0.0084
fo_Decile_60	-0.0281	0.1029	-0.0329	-0.0826	0.0013
fo_Decile_70	0.0364	0.0881	-0.0543	-0.0568	0.0126
fo_Decile_90	0.0748	-0.0469	0.0016	0.0615	0.0106

glcm_Contrast	0.0072	0.0380	0.1351	0.0075	0.0212
glcm_Contrast_s	-0.0118	0.0136	0.1463	0.0179	0.0573
glcm_Contrast_e	0.0026	0.0398	0.1281	0.0068	-0.0005
glcm_Homogeneity2	-0.0411	-0.0365	-0.1325	-0.0085	-0.0080
glcm_Homogeneity2_s	-0.0796	-0.0452	-0.0534	-0.0152	0.0129
glcm_Homogeneity2_e	-0.0024	-0.0217	-0.1519	-0.0049	-0.0177
glcm_Homogeneity2_nd	-0.0397	-0.0340	-0.1346	-0.0082	-0.0102
glcm_Homogeneity2_s_nd	-0.0829	-0.0448	-0.0428	-0.0133	0.0085
glcm_Homogeneity2_e_nd	0.0067	-0.0154	-0.1522	-0.0053	-0.0182
glcm_Dissimilarity	0.0267	0.0358	0.1411	0.0077	0.0169
glcm_Dissimilarity_s	-0.0682	-0.0183	0.0952	0.0062	0.0327
glcm_Dissimilarity_e	0.0319	0.0397	0.1324	0.0050	-0.0034
glcm_Homogeneity1	-0.0401	-0.0362	-0.1345	-0.0085	-0.0091
glcm_Homogeneity1_s	-0.0819	-0.0450	-0.0457	-0.0145	0.0133
glcm_Homogeneity1_e	0.0158	-0.0139	-0.1511	-0.0048	-0.0233
glcm_Homogeneity1_nd	-0.0358	-0.0315	-0.1369	-0.0080	-0.0136
glcm_Homogeneity1_s_nd	-0.0858	-0.0436	-0.0280	-0.0120	0.0095
glcm_Homogeneity1_e_nd	0.0444	0.0035	-0.1329	-0.0045	-0.0273
glcm_DMN	0.0072	0.0380	0.1351	0.0075	0.0212
glcm_DMN_e	0.0026	0.0398	0.1281	0.0068	-0.0005
glcm_IDMN	-0.0137	-0.0362	-0.1379	-0.0068	-0.0210
glcm_IDMN_s	-0.0868	-0.0430	-0.0201	-0.0115	0.0144
glcm_IDMN_e	0.0893	0.0366	-0.0029	0.0003	-0.0316
glcm_IDMN_nd	0.0463	0.0352	0.1144	0.0083	0.0009
glcm_IDMN_s_nd	-0.0881	-0.0404	-0.0010	-0.0088	0.0128
glcm_IDMN_e_nd	0.0840	0.0419	0.0463	0.0017	-0.0229
glcm_DN	0.0267	0.0358	0.1411	0.0077	0.0169
glcm_DN_s	-0.0682	-0.0183	0.0952	0.0062	0.0327
glcm_DN_e	0.0319	0.0397	0.1324	0.0050	-0.0034
glcm_IDN	-0.0312	-0.0350	-0.1405	-0.0078	-0.0153
glcm_IDN_s	-0.0865	-0.0433	-0.0229	-0.0119	0.0140
glcm_IDN_e	0.0878	0.0329	-0.0312	-0.0005	-0.0342
glcm_IDN_nd	0.0449	0.0340	0.0908	0.0078	-0.0055
glcm_IDN_s_nd	-0.0880	-0.0408	-0.0043	-0.0093	0.0123
glcm_IDN_e_nd	0.0865	0.0415	0.0287	0.0013	-0.0255
glcm_Autocorrelation	-0.0498	0.1129	-0.0409	0.0182	0.0067
glcm_Autocorrelation_s	-0.0908	0.0114	-0.0174	-0.0323	0.0100
glcm_Autocorrelation_e	0.0049	0.1273	-0.0476	0.0633	-0.0035
glcm_Autocorrelation_nd	-0.0374	0.1259	-0.0137	0.0316	0.0112
glcm_Autocorrelation_s_nd	-0.0911	0.0173	-0.0045	-0.0302	0.0087
glcm_Autocorrelation_e_nd	0.0188	0.1267	-0.0187	0.0739	0.0010
glcm_Inv_autocorrelation	0.0560	-0.0208	-0.0126	-0.1737	0.0238
glcm_Inv_autocorrelation_e	0.0439	0.0220	-0.0090	-0.1938	-0.0028

glcm_Inv_autocorrelation_nd	0.0554	-0.0070	0.0099	-0.1772	0.0247
glcm_Inv_autocorrelation_e_nd	0.0417	0.0298	0.0048	-0.1936	-0.0016
glcm_Gauss	-0.0869	-0.0227	0.0271	0.0026	-0.0443
glcm_Gauss_s	-0.0884	-0.0379	-0.0125	-0.0122	0.0072
glcm_Gauss_e	-0.0065	0.0275	0.1046	0.0228	-0.1489
glcm_Gauss_nd	-0.0665	-0.0054	0.0934	0.0097	-0.0452
glcm_Gauss_s_nd	-0.0890	-0.0353	0.0048	-0.0095	0.0049
glcm_Gauss_e_nd	0.0194	0.0339	0.1329	0.0189	-0.0830
glcm_Gauss_lp	0.0786	-0.0705	-0.0224	-0.0216	0.0170
glcm_Gauss_lp_s	0.0306	-0.1208	-0.0334	0.0432	0.0295
glcm_Gauss_lp_e	0.0822	-0.0533	-0.0216	-0.0489	0.0086
glcm_Gauss_lp_nd	0.0802	-0.0668	-0.0132	-0.0290	0.0170
glcm_Gauss_lp_s_nd	0.0353	-0.1214	-0.0194	0.0437	0.0289
glcm_Gauss_lp_e_nd	0.0827	-0.0492	-0.0138	-0.0578	0.0081
glcm_Gauss_lf	0.0629	-0.1039	-0.0029	0.0107	0.0023
glcm_Gauss_lf_s	-0.0348	-0.1209	-0.0244	0.0340	0.0197
glcm_Gauss_lf_e	0.0772	-0.0782	0.0026	-0.0108	-0.0073
glcm_Gauss_lf_nd	0.0668	-0.0963	0.0184	0.0059	0.0017
glcm_Gauss_lf_s_nd	-0.0313	-0.1250	-0.0035	0.0384	0.0182
glcm_Gauss_lf_e_nd	0.0787	-0.0711	0.0203	-0.0168	-0.0080
glcm_Gauss_rf	-0.0592	0.1045	-0.0317	-0.0121	-0.0027
glcm_Gauss_rf_s	-0.0893	0.0242	-0.0160	-0.0386	0.0073
glcm_Gauss_rf_e	-0.0280	0.1284	-0.0399	0.0146	-0.0100
glcm_Gauss_rf_nd	-0.0525	0.1148	-0.0153	-0.0045	-0.0006
glcm_Gauss_rf_s_nd	-0.0891	0.0298	-0.0054	-0.0368	0.0058
glcm_Gauss_rf_e_nd	-0.0179	0.1346	-0.0215	0.0247	-0.0073
glcm_Gauss_rp	-0.0124	0.1188	-0.0788	0.0230	0.0242
glcm_Gauss_rp_s	-0.0779	0.0617	-0.0299	-0.0474	0.0151
glcm_Gauss_rp_e	0.0155	0.1080	-0.0878	0.0554	0.0185
glcm_Gauss_rp_nd	-0.0052	0.1221	-0.0719	0.0318	0.0284
glcm_Gauss_rp_s_nd	-0.0768	0.0682	-0.0232	-0.0452	0.0150
glcm_Gauss_rp_e_nd	0.0221	0.1072	-0.0801	0.0635	0.0222
glcm_Inv_Gauss	0.0778	0.0314	-0.0447	-0.0159	0.0615
glcm_Inv_Gauss_s	-0.0834	-0.0492	-0.0306	-0.0107	0.0295
glcm_Inv_Gauss_e	0.0817	0.0393	-0.0260	-0.0158	0.0124
glcm_Inv_Gauss_nd	0.0798	0.0426	0.0081	-0.0148	0.0592
glcm_Inv_Gauss_s_nd	-0.0856	-0.0467	-0.0082	-0.0079	0.0311
glcm_Inv_Gauss_e_nd	0.0808	0.0449	0.0034	-0.0168	0.0142
glcm_Inv_Gauss_lp	0.0154	-0.0198	-0.0802	0.1127	0.0269
glcm_Inv_Gauss_lp_s	-0.0525	0.0808	-0.0751	0.0035	0.0682
glcm_Inv_Gauss_lp_e	0.0157	-0.0302	-0.0753	0.1116	0.0109
glcm_Inv_Gauss_lp_nd	0.0213	-0.0185	-0.0792	0.1160	0.0330

glcm_Inv_Gauss_lp_s_nd	-0.0472	0.0914	-0.0699	0.0086	0.0765
glcm_Inv_Gauss_lp_e_nd	0.0207	-0.0296	-0.0748	0.1142	0.0159
glcm_Inv_Gauss_lf	0.0228	0.0353	-0.0913	0.1193	0.0448
glcm_Inv_Gauss_lf_s	-0.0833	0.0440	-0.0375	-0.0303	0.0306
glcm_Inv_Gauss_lf_e	0.0333	0.0088	-0.0816	0.1250	0.0221
glcm_Inv_Gauss_lf_nd	0.0306	0.0359	-0.0794	0.1237	0.0506
glcm_Inv_Gauss_lf_s_nd	-0.0826	0.0534	-0.0263	-0.0270	0.0333
glcm_Inv_Gauss_lf_e_nd	0.0386	0.0088	-0.0728	0.1269	0.0266
glcm_Inv_Gauss_rf	0.0671	-0.0148	-0.0246	-0.1507	0.0282
glcm_Inv_Gauss_rf_s	0.0096	-0.1275	-0.0398	0.0229	0.0459
glcm_Inv_Gauss_rf_e	0.0545	0.0154	-0.0227	-0.1799	0.0064
glcm_Inv_Gauss_rf_nd	0.0652	-0.0061	-0.0123	-0.1591	0.0284
glcm_Inv_Gauss_rf_s_nd	0.0162	-0.1301	-0.0196	0.0231	0.0478
glcm_Inv_Gauss_rf_e_nd	0.0514	0.0214	-0.0141	-0.1843	0.0062
glcm_Inv_Gauss_rp	0.0341	0.0282	-0.0313	-0.1993	0.0223
glcm_Inv_Gauss_rp_s	0.0569	-0.0957	-0.0411	-0.0028	0.0553
glcm_Inv_Gauss_rp_e	0.0222	0.0406	-0.0296	-0.2036	0.0038
glcm_Inv_Gauss_rp_nd	0.0336	0.0308	-0.0277	-0.1990	0.0228
glcm_Inv_Gauss_rp_s_nd	0.0611	-0.0937	-0.0301	-0.0072	0.0566
glcm_Inv_Gauss_rp_e_nd	0.0222	0.0419	-0.0274	-0.2026	0.0047
glcm_Gauss_2f	0.0314	-0.0256	-0.1375	-0.0023	-0.0008
glcm_Gauss_2f_s	-0.0858	-0.0452	-0.0249	-0.0114	0.0158
glcm_Gauss_2f_e	0.0873	0.0245	-0.0411	-0.0001	-0.0225
glcm_Gauss_2f_nd	0.0766	0.0205	0.0182	0.0070	0.0044
glcm_Gauss_2f_s_nd	-0.0875	-0.0428	-0.0061	-0.0088	0.0143
glcm_Gauss_2f_e_nd	0.0891	0.0333	0.0067	0.0011	-0.0178
glcm_Inv_Gauss_2f	0.0657	0.0152	-0.0852	-0.0220	0.0536
glcm_Inv_Gauss_2f_s	-0.0682	-0.0590	-0.0650	-0.0097	0.0634
glcm_Inv_Gauss_2f_e	0.0644	0.0177	-0.0804	-0.0266	0.0220
glcm_Inv_Gauss_2f_nd	0.0702	0.0218	-0.0671	-0.0261	0.0578
glcm_Inv_Gauss_2f_s_nd	-0.0714	-0.0587	-0.0444	-0.0077	0.0765
glcm_Inv_Gauss_2f_e_nd	0.0672	0.0223	-0.0676	-0.0312	0.0255
glcm_Gauss_2p	0.0773	0.0102	-0.0828	-0.0068	0.0366
glcm_Gauss_2p_s	-0.0602	-0.0664	-0.0764	-0.0076	0.0534
glcm_Gauss_2p_e	0.0815	0.0179	-0.0715	-0.0098	0.0186
glcm_Gauss_2p_nd	0.0820	0.0165	-0.0659	-0.0082	0.0387
glcm_Gauss_2p_s_nd	-0.0630	-0.0683	-0.0607	-0.0055	0.0617
glcm_Gauss_2p_e_nd	0.0839	0.0218	-0.0596	-0.0117	0.0202
glcm_Inv_Gauss_2p	0.0366	-0.0003	-0.0955	-0.0233	0.0397
glcm_Inv_Gauss_2p_s	0.0185	-0.0381	-0.1175	0.0000	0.1279
glcm_Inv_Gauss_2p_e	0.0303	-0.0024	-0.0938	-0.0273	0.0132
glcm_Inv_Gauss_2p_nd	0.0425	0.0041	-0.0915	-0.0297	0.0459
glcm_Inv_Gauss_2p_s_nd	0.0311	-0.0293	-0.1056	-0.0008	0.1453

glcm_Inv_Gauss_2p_e_nd	0.0358	0.0012	-0.0915	-0.0339	0.0186
glcm_Cluster_p	-0.0213	0.1219	-0.0647	0.0344	0.0244
glcm_Cluster_p_s	-0.0848	0.0443	-0.0226	-0.0421	0.0129
glcm_Cluster_p_e	0.0168	0.1106	-0.0726	0.0758	0.0170
glcm_Cluster_p_nd	-0.0113	0.1272	-0.0516	0.0464	0.0299
glcm_Cluster_p_s_nd	-0.0841	0.0509	-0.0133	-0.0399	0.0126
glcm_Cluster_p_e_nd	0.0259	0.1086	-0.0583	0.0855	0.0213
glcm_Inv_Cluster_p_e	-0.0172	0.0353	-0.0105	-0.1410	-0.0145
glcm_Inv_Cluster_p_e_nd	-0.0053	0.0479	-0.0170	-0.1786	-0.0025
glcm_Cluster_s	-0.0359	0.1212	-0.0492	0.0245	0.0162
glcm_Cluster_s_s	-0.0884	0.0296	-0.0190	-0.0381	0.0110
glcm_Cluster_s_e	0.0085	0.1221	-0.0568	0.0683	0.0085
glcm_Cluster_s_nd	-0.0250	0.1298	-0.0305	0.0371	0.0214
glcm_Cluster_s_s_nd	-0.0881	0.0360	-0.0080	-0.0360	0.0102
glcm_Cluster_s_e_nd	0.0197	0.1211	-0.0369	0.0790	0.0128
glcm_Inv_Cluster_s_e	0.0140	0.0306	-0.0209	-0.1888	-0.0077
glcm_Inv_Cluster_s_e_nd	0.0296	0.0316	-0.0199	-0.1973	0.0008
glcm_Cluster_t	-0.0488	0.1157	-0.0335	0.0191	0.0080
glcm_Cluster_t_s	-0.0909	0.0116	-0.0168	-0.0322	0.0102
glcm_Cluster_t_e	0.0060	0.1295	-0.0366	0.0637	-0.0034
glcm_Cluster_t_nd	-0.0357	0.1277	-0.0043	0.0322	0.0127
glcm_Cluster_t_s_nd	-0.0911	0.0176	-0.0037	-0.0301	0.0091
glcm_Cluster_t_e_nd	0.0195	0.1274	-0.0067	0.0732	0.0011
glcm_Inv_Cluster_t	0.0729	-0.0520	-0.0206	-0.1091	0.0187
glcm_Inv_Cluster_t_e	0.0709	-0.0118	-0.0171	-0.1443	-0.0013
glcm_Inv_Cluster_t_nd	0.0763	-0.0422	0.0014	-0.1095	0.0197
glcm_Inv_Cluster_t_e_nd	0.0723	-0.0058	-0.0017	-0.1425	0.0002
glcm_Cluster_d	-0.0594	0.1067	-0.0189	0.0195	0.0004
glcm_Cluster_d_s	-0.0913	-0.0116	-0.0166	-0.0236	0.0112
glcm_Cluster_d_e	0.0272	0.1277	-0.0094	0.0591	-0.0219
glcm_Cluster_d_nd	-0.0367	0.1219	0.0379	0.0332	0.0053
glcm_Cluster_d_s_nd	-0.0920	-0.0066	-0.0006	-0.0214	0.0097
glcm_Cluster_d_e_nd	0.0395	0.1157	0.0358	0.0603	-0.0145
glcm_Inv_Cluster_d	0.0740	-0.0796	-0.0071	-0.0497	0.0132
glcm_Inv_Cluster_d_s	-0.0715	-0.0825	-0.0252	0.0049	0.0218
glcm_Inv_Cluster_d_e	0.0874	-0.0236	0.0024	-0.0677	-0.0100
glcm_Inv_Cluster_d_nd	0.0786	-0.0566	0.0387	-0.0515	0.0132
glcm_Inv_Cluster_d_s_nd	-0.0720	-0.0842	-0.0026	0.0086	0.0210
glcm_Inv_Cluster_d_e_nd	0.0862	-0.0115	0.0306	-0.0692	-0.0091
glcm_Average	-0.0599	0.1061	-0.0186	0.0192	0.0003
glcm_Average_s	-0.0913	-0.0117	-0.0165	-0.0236	0.0112
glcm_Average_e	0.0266	0.1280	-0.0092	0.0591	-0.0221
glcm_Variances	0.0859	0.0267	-0.0331	-0.0059	0.0465

glcm_Variances_s	-0.0909	0.0120	-0.0159	-0.0321	0.0104
glcm_Variances_e	0.0542	0.1094	-0.0008	0.0435	-0.0271
glcm_Correlation	0.0545	-0.0096	-0.1235	-0.0089	0.0129
glcm_Correlation_s	-0.0733	0.0051	-0.0874	-0.0255	-0.0243
glcm_Correlation_e	0.0291	0.0258	-0.1277	0.0203	-0.0196
glcm_Sum_average	-0.0599	0.1061	-0.0186	0.0192	0.0003
glcm_Sum_energy	-0.0873	0.0103	0.0490	-0.0109	-0.0099
glcm_Sum_entropy	0.0813	0.0238	-0.0701	-0.0116	0.0152
glcm_Sum_variance	-0.0573	0.1083	-0.0241	0.0193	0.0068
glcm_Dif_average	0.0267	0.0358	0.1411	0.0077	0.0169
glcm_Dif_entropy	0.0186	0.0332	0.1430	0.0066	0.0131
glcm_Dif_variance	-0.0208	0.0351	0.1093	0.0054	0.0238
glcm_Inv_sum_average	0.0742	-0.0790	-0.0074	-0.0497	0.0132
glcm_Inv_sum_energy	-0.0647	-0.0725	0.0696	0.0186	-0.0088
glcm_Inv_sum_variance	-0.0046	0.0114	-0.0100	-0.0780	0.0210
glcm_Inv_dif_average	-0.0383	-0.0332	-0.1360	-0.0081	-0.0116
glcm_Inv_dif_variance	0.0097	-0.0007	-0.0450	0.0030	-0.0162
glcm_IMC1	0.0789	0.0394	0.0566	0.0038	-0.0709
glcm_IMC2	0.0241	0.0070	-0.0309	-0.0088	-0.2421
glcm_Energy	-0.0869	-0.0430	-0.0192	-0.0114	0.0147
glcm_Entropy	0.0255	0.0075	-0.0312	-0.0088	-0.2409
glcm_Median	0.0136	0.0025	0.0127	-0.0451	-0.1009
glcm_Geo_mean	-0.0142	0.0008	0.0374	0.0097	0.2507
glcm_Geo_mean2	-0.0142	0.0008	0.0374	0.0097	0.2507
glcm_Har_mean	0.0021	0.0130	0.0526	-0.0009	0.2544
glcm_Trim_mean_5	0.0853	0.0456	0.0118	0.0089	-0.0117
glcm_Trim_mean_10	0.0878	0.0418	0.0110	0.0052	-0.0029
glcm_Trim_mean_20	0.0880	0.0369	0.0160	0.0008	0.0069
glcm_IQ_mean	0.0772	0.0365	0.0266	-0.0225	-0.0238
glcm_Tri_mean	0.0136	0.0025	0.0127	-0.0451	-0.1009
glcm_Mn_AD_mn	-0.0806	-0.0352	-0.0299	0.0146	0.0140
glcm_Md_AD_md	0.0136	0.0025	0.0127	-0.0451	-0.1009
glcm_MAD	0.0136	0.0025	0.0127	-0.0451	-0.1009
glcm_Max_AD_mn	-0.0751	-0.0484	-0.0201	-0.0144	0.0397
glcm_Max_AD_md	-0.0752	-0.0484	-0.0203	-0.0138	0.0410
glcm_RMS	-0.0872	-0.0432	-0.0172	-0.0093	0.0135
glcm_Max	-0.0751	-0.0484	-0.0201	-0.0144	0.0397
glcm_Quartiles	0.0828	0.0253	0.0283	0.0068	0.0534
glcm_IQR	0.0828	0.0253	0.0283	0.0068	0.0534
glcm_Low_notch	-0.0828	-0.0253	-0.0283	-0.0068	-0.0534
glcm_High_notch	0.0828	0.0253	0.0283	0.0068	0.0534
glcm_Range	-0.0751	-0.0484	-0.0201	-0.0144	0.0397
glcm_Deciles	0.0878	0.0383	0.0094	0.0016	0.0076

glcm_SD	-0.0872	-0.0433	-0.0168	-0.0089	0.0133
glcm_Skew	-0.0760	-0.0471	-0.0114	-0.0138	0.0274
glcm_Kurtosis	-0.0705	-0.0458	-0.0155	-0.0176	0.0393
glcm_Uniformity	-0.0395	-0.0093	0.0328	0.0025	0.2279
glrlm_SRE	0.0357	0.0405	0.1323	0.0001	0.0416
glrlm_LRE	-0.0354	-0.0371	-0.1296	-0.0024	-0.0387
glrlm_GLN	-0.0188	-0.0171	-0.0416	0.0107	-0.2414
glrlm_RLN	0.0230	-0.0034	-0.0431	0.0138	-0.2375
glrlm_RP	0.0364	0.0400	0.1311	0.0017	0.0412
glrlm_LGLRE	-0.0556	0.0663	0.0066	-0.0920	0.0140
glrlm_HGLRE	0.0147	0.0938	-0.0407	0.0232	0.0550
glrlm_SRLGLE	-0.0558	0.0674	0.0090	-0.0901	0.0145
glrlm_LRHGLE	-0.0406	0.0650	-0.1065	-0.0039	-0.0015
glrlm_SRHGLE	0.0328	0.0855	-0.0092	0.0284	0.0659
glrlm_LRLGLE	-0.0544	0.0593	-0.0082	-0.1002	0.0112

Supplementary Table 2 | Statistics for pairwise models of baseline group differences in texture and volume between all group combinations. P-values here are adjusted across all 36 tests using Bonferroni-Holm false discovery rate correction. CU-Aβ- = Cognitively unimpaired amyloid negative group; CU-Aβ+ = Cognitively unimpaired amyloid positive group; MCI-Aβ+ = Mild cognitive impairment (amyloid positive) group; ADD-Aβ+ = Alzheimer's disease dementia (amyloid positive) group

	F	df1	df2	Cohen's *d*	p_{adj}
**Tx PC1**					
CU-Aβ- vs CU-Aβ+	0.21	1	573	0.04	0.698
CU-Aβ- vs MCI-Aβ+	21.2	1	710	0.35	<.0001
CU-Aβ- vs ADD-Aβ+	37.2	1	564	0.51	<.0001
CU-Aβ+ vs MCI-Aβ+	8.13	1	535	0.25	0.007
CU-Aβ+ vs ADD-Aβ+	17.6	1	382	0.43	<.0001
MCI-Aβ+ vs ADD-Aβ+	3.42	1	514	0.16	0.094
**Tx PC2**					
CU-Aβ- vs CU-Aβ+	3.26	1	577	0.15	0.096
CU-Aβ- vs MCI-Aβ+	<0.01	1	690	<0.01	0.990
CU-Aβ- vs ADD-Aβ+	11.2	1	492	0.30	0.002
CU-Aβ+ vs MCI-Aβ+	3.24	1	501	0.16	0.096
CU-Aβ+ vs ADD-Aβ+	18.8	1	346	0.47	<.0001
MCI-Aβ+ vs ADD-Aβ+	8.88	1	469	0.28	0.005
**Tx PC3**					
CU-Aβ- vs CU-Aβ+	1.09	1	545	0.09	0.372
CU-Aβ- vs MCI-Aβ+	0.98	1	672	0.08	0.384
CU-Aβ- vs ADD-Aβ+	0.34	1	536	0.05	0.630
CU-Aβ+ vs MCI-Aβ+	3.9	1	510	0.17	0.074
CU-Aβ+ vs ADD-Aβ+	<0.01	1	373	<0.01	0.990
MCI-Aβ+ vs ADD-Aβ+	1.07	1	486	0.09	0.372
**Tx PC4**					
CU-Aβ- vs CU-Aβ+	7.21	1	564	0.23	0.012
CU-Aβ- vs MCI-Aβ+	39	1	695	0.47	<.0001
CU-Aβ- vs ADD-Aβ+	111	1	547	0.90	<.0001
CU-Aβ+ vs MCI-Aβ+	8.21	1	526	0.25	0.007
CU-Aβ+ vs ADD-Aβ+	45.4	1	374	0.70	<.0001
MCI-Aβ+ vs ADD-Aβ+	22.6	1	502	0.42	<.0001
**Tx PC5**					
CU-Aβ- vs CU-Aβ+	0.59	1	558	0.07	0.511
CU-Aβ- vs MCI-Aβ+	47.4	1	685	0.53	<.0001
CU-Aβ- vs ADD-Aβ+	142	1	519	1.05	<.0001
CU-Aβ+ vs MCI-Aβ+	41.1	1	515	0.56	<.0001
CU-Aβ+ vs ADD-Aβ+	108	1	345	1.12	<.0001
MCI-Aβ+ vs ADD-Aβ+	39.3	1	456	0.59	<.0001
**Volume**					
CU-Aβ- vs CU-Aβ+	0.14	1	574	0.03	0.752
CU-Aβ- vs MCI-Aβ+	49.9	1	715	0.53	<.0001
CU-Aβ- vs ADD-Aβ+	210	1	565	1.22	<.0001
CU-Aβ+ vs MCI-Aβ+	31	1	541	0.48	<.0001
CU-Aβ+ vs ADD-Aβ+	131	1	388	1.16	<.0001
MCI-Aβ+ vs ADD-Aβ+	67.3	1	519	0.72	<.0001

Speculative interpretation of texture components.

We have provided a description in the main discussion as to what texture principal component 4 (PC4) may represent. Other components are more speculative, but we have attempted to describe them here.

Note 1: GLCM = Grey-Level Co-Occurrence Matrix.
Note 2: A full description of every mathematical formula behind each texture variable is provided by Kolossváry et al., 2017, *Circulation. Cardiovascular Imaging*, *10*(12).

PC1

PC1 appears to be a measure of image 'randomness', with high values of PC1 constituting more random texture patterns across the ROI. Strong negative loadings of average squared-GLCM (glcm_Average_s) and squared-GLCM variance (glcm_Variances_s) suggest uniform values throughout the GLCM. In other words, low-intensity value voxels are similarly likely to appear adjacent to high-intensity value voxels as other low-intensity voxels, indicative of a random spread of values. This first PC may absorb a large source of noise variation in the dataset: that which is caused by image noise and movement artefacts. Whilst potentially relevant in terms of differences between healthy controls and people with Alzheimer's disease, this component may lack a specific biological cause.

PC2

PC2 appears to be primarily determined by clustering. GLCM measures for clustering load positively in PC2, including clustering difference, tendency, shade and prominence. Negative loading of gaussian left focus (glcm_Gauss_lf[_s][_nd]) and left polar (glcm_Gauss_lp[_s][_nd]) indicates fewer high intensity voxels adjacent to other high intensity voxels in those with high PC2, while negative loading of inverse gaussian right focus (glcm_Inv_Gauss_rf[_s][_nd]) indicates a higher proportion of voxel pairs that are not both low intensity. Together, this suggests weighting towards off-diagonal pairings (adjacent high-low intensity voxels). Biologically, this could suggest presence of cysts, sclerotic lesions, or differences in the proportions of grey to white matter within the ROI.

PC3

PC3 is likely sensitive to patches of heterogeneity, with higher values indicating high probability of patches with high heterogeneity / low homogeneity. This is indicated with strong positive loadings of GLCM dissimilarity and contrast combined with negative loadings of multiple measures of GLCM homogeneity. This is in contrast to PC5 where most measures loading are first-order statistics, and are therefore indicative of uniform homogeneity across the entire ROI, PC3 is more characterized by localized patches of this texture.

PC4

The factors that load strongly onto PC4 (e.g. negative loading of inverse right-polar gaussian and inverse autocorrelation GLCM factors) indicate that a 'high-PC4' hippocampus possesses

a higher proportion of high-intensity voxels appearing adjacent to other high-intensity voxels. In other words, these cases display quantifiable clustering of hyperintense areas. This may indicate localized increases in CSF due to small amounts of atrophy or accumulation of paramagnetic materials. Future studies could characterize how microstructural changes, for example neuroinflammation or blood vessel damage, affect conceptually interpretable changes in texture features. (From main discussion).

PC5

PC5 may represent homogeneity of the overall ROI. High positive loading of first-order statistics entropy and uniformity, and negative loading of first-order energy and root-mean-square indicate that a high-PC5 hippocampus is generally more homogenous than a low PC5 hippocampus. PC5 is the texture component most closely related to volume (negative correlation r=-0.49, Fig 2B). Some variation in PC5 may be directly caused by variation in the number of voxels in the image (lower volume means fewer voxels which is related to greater homogeneity).
